# FTO depletion does not alter m^6^A stoichiometry in AML mRNA: a reassessment using direct RNA nanopore sequencing

**DOI:** 10.1101/2025.10.22.681652

**Published:** 2025-10-23

**Authors:** Luke S. Nicholson, Catarina Guimarães-Teixeira, Jianheng Fox Liu, Hui Xian Poh, Samie R. Jaffrey

**Affiliations:** 1 Department of Pharmacology, Weill Cornell Medicine, Cornell University, New York, NY 10065, USA; 2 Cancer Biology and Epigenetics Group, Research Center of IPO Porto (CI-IPOP)/RISE@CI-IPOP (Health Research Network), Portuguese Oncology Institute of Porto (IPO Porto)/Porto Comprehensive Cancer Center Raquel Seruca (Porto.CCC Raquel Seruca), Porto, Portugal; 3 A*STAR Infectious Diseases Labs (A*STAR ID Labs), Agency for Science, Technology and Research (A*STAR), Singapore 138648, Singapore

## Abstract

The RNA demethylase FTO has been proposed to promote acute myeloid leukemia (AML) by demethylating *N*^6^-methyladenosine (m^6^A) from oncogenic transcripts, especially *MYC*. However, the evidence that supports the idea that FTO demethylates m^6^A in AML relies on methods that are non-quantitative and unable to reveal m^6^A stoichiometry changes before or after FTO depletion. To directly test whether FTO regulates m^6^A in mRNA, we employed Oxford Nanopore direct RNA sequencing to map and quantify m^6^A at single-nucleotide resolution. We find that the stoichiometry of m^6^A sites throughout the transcriptome and especially at *MYC*-specific sites are unaffected despite depletion of FTO activity by knockout, knockdown, or pharmacologic inhibition. This pattern was seen in AML cell lines MONOMAC-6 and MOLM-13, as well as in the non-AML cell line HEK293T. We also find that the anti-leukemia effect of the small-molecule FTO inhibitor FB23–2 is not due to FTO inhibition since it remains cytotoxic to FTO-deficient cells. Instead of regulating m^6^A, we find that FTO depletion markedly increases *N*^6^,2’-*O*-dimethyladenosine (m^6^Am) in snRNAs, consistent with m^6^Am in snRNA being a target of FTO. Overall, our findings do not support an ‘m^6^A eraser’ role for FTO in AML cell lines under the conditions tested, and they suggest that the reported demethylation functions of FTO on m^6^A should be reinvestigated using quantitative m^6^A mapping methods.

## INTRODUCTION

FTO (fat mass and obesity-associated protein) is an RNA demethylase that has roles in diverse cellular and physiological processes^1,2^. FTO is thought to mediate its effects by demethylating methylated nucleotides in RNA, thus influencing stability, translation, or other aspects of RNA metabolism^1^. The initial study that linked FTO to RNA demethylation showed that FTO demethylates m^6^A, the most prevalent internal modification in mRNA^3^. Subsequent work focused on identifying whether FTO demethylates all or only some m^6^A sites, and on determining how the demethylation of m^6^A explains specific cellular and physiologic effects of FTO^1,2^.

Although FTO was originally shown to demethylate m^6^A, we previously noted that FTO exhibits a low catalytic rate of demethylation on m^6^A when compared to enzymatic reactions performed by other a-ketoglutarate-dependent dioxygenases^4^. Our transcriptome-wide mapping studies in *FTO* knockout brain showed no clear increases in m^6^A sites^5^; in contrast, we observed clear increases in *N*^6^,2′-*O*-dimethyladenosine (m^6^Am)^4^. In contrast to m^6^A, which is located inside the transcript body, m^6^Am is located at the first transcribed nucleotide of snRNAs, snoRNAs, and mRNAs^4,6,7^. m^6^Am is similar to m^6^A but contains an additional methyl modification on the 2’ position of the ribose and also reacts with m^6^A antibodies used in mapping studies^4,8^. We found that FTO primarily demethylates m^6^Am in snRNAs, with much more subtle activity towards m^6^Am in mRNA^6,7^.

However, despite the clear ability of FTO to demethylate m^6^Am, a series of subsequent studies showed that FTO also demethylates m^6^A in cells, and that m^6^A demethylation accounts for the main aspects of FTO biology, especially its ability to promote cancer. These studies focused on the demethylation activity of FTO in acute myeloid leukemia (AML). The initial study by Li et al. reported that FTO is an oncogene in AML and promotes leukemogenesis by demethylating m^6^A on key transcripts, including *ASB2* and *RARA*^9^. Building on this, Su et al. showed that the oncometabolite R-2-hydroxyglutarate suppresses the demethylase function of FTO, leading to increased m^6^A levels and impaired AML cell growth^10^. Su et al. also showed that FTO demethylates m^6^A sites in *MYC*, a master transcriptional regulator in cancer. FTO-mediated m^6^A demethylation of *MYC* leads to stabilization of *MYC* and increased *MYC* expression, thus acting as a major oncogenic mechanism in AML and potentially many other cancers^10^. Huang et al. developed FB23–2, a small-molecule FTO inhibitor that increased m^6^A levels on *MYC* and other AML-relevant transcripts and demonstrated potent anti-leukemic effects in both cell lines and xenograft models^11^. These major studies have led to follow-up studies that used similar approaches to report that FTO demethylates functionally important m^6^A sites in different cancers and other disease contexts^12–14^.

These studies mapped FTO targets using MeRIP/m^6^A-seq, a transcriptome-wide m^6^A mapping method developed by us and others^15,16^. MeRIP-seq uses m^6^A antibodies to immunoprecipitate m^6^A-containing RNA fragments for sequencing^15^. The resulting peaks can exhibit considerable variability in peak heights, even between biological replicates, because RNA precipitation efficiency is highly sensitive to subtle changes in washing and other immunoprecipitation conditions^17^. This is important because targets of FTO were identified based on differential peak heights that occur after FTO depletion. Thus, these peak height differences might not reflect changes in m^6^A levels, but instead could simply be the normal peak height variability in MeRIP-seq.

In order to know if an m^6^A site in *MYC* and other transcripts is indeed regulated by FTO, its stoichiometry should be measured before and after FTO depletion. The stoichiometry of an m^6^A site refers to the fraction of transcripts that contain m^6^A at that site. An m^6^A site that is normally demethylated by FTO should have low stoichiometry under control conditions and increased m^6^A stoichiometry once FTO is depleted. Since MeRIP-seq does not provide single-nucleotide resolution and is not quantitative, the magnitude of the stoichiometry changes in methylation achieved by FTO depletion could not be reported in the AML studies or in subsequent studies of FTO and m^6^A.

To discover the m^6^A sites in *MYC* and other mRNAs that are regulated by FTO, we performed quantitative transcriptome-wide m^6^A mapping using Oxford Nanopore direct RNA sequencing^18^. We measured m^6^A sites in the AML cell lines MONOMAC-6 and MOLM-13, as well as in HEK293T cells, under conditions of *FTO* knockdown, *FTO* knockout, and pharmacologic FTO inhibition. Contrary to expectations based on the published studies, we did not observe changes in m^6^A stoichiometry in any of the measurable sites in the cell lines and conditions tested. This includes m^6^A sites in *MYC* and other proposed FTO targets. In addition, we found that *FTO* knockdown did not affect cell viability, suggesting that FTO is not absolutely required for AML survival. We also found that the FTO inhibitor FB23–2 remained cytotoxic even in FTO-deficient cells, suggesting that this inhibitor mediates its anti-leukemic effects in an FTO-independent manner. Overall, our data highlight the importance of re-examining FTO’s proposed ‘m^6^A eraser’ role using quantitative, site-specific assays across diverse cellular contexts.

## RESULTS

### Nanopore direct RNA sequencing provides highly accurate m^6^A stoichiometry measurements

To identify the sites in the AML transcriptome that are regulated by FTO, we chose to use Oxford Nanopore direct RNA sequencing of poly(A) RNA. Recent improvements in the nanopore technology and flow cell chemistry (“RNA004”) have resulted in markedly more accurate detection of m^6^A in single RNA reads^19,20^. m^6^A is detected using the Dorado basecaller, a neural network trained on raw nanopore current signals to assign per-read modification probabilities^21^. When multiple independent mRNAs for a specific gene are sequenced, the stoichiometry of m^6^A at any site within the mRNA can be calculated^21–23^.

Recent studies have extensively validated the ability of Nanopore sequencing to accurately measure m^6^A stoichiometry by comparing Nanopore-predicted m^6^A stoichiometries in cellular RNA at sites with known stoichiometry. These comparisons are performed using a dataset of m^6^A stoichiometries measured using GLORI, a highly accurate and quantitative m^6^A mapping method that uses a chemical approach to distinguish A from m^6^A on a transcriptome-wide level^24^. Recent studies have extensively documented that m^6^A stoichiometry measurements made using the newest RNA004 flow cell chemistry result in m^6^A stoichiometries that are very similar to those measured using GLORI^25,26^. In these experiments, thousands of m^6^A sites with known stoichiometries, as measured by GLORI in HEK293 cells, are compared to the stoichiometries measured for those same sites by Nanopore sequencing. These comparisons of m^6^A stoichiometry by Nanopore versus GLORI across thousands of sites validate the Nanopore technology in a massively parallel manner. A benefit of Nanopore sequencing is that it also detects m^6^A in long-reads, which is not possible with GLORI.

**Figure 1.**
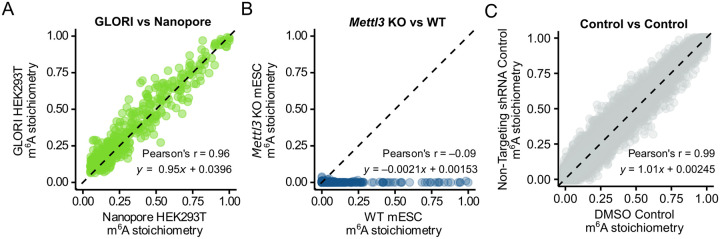
Nanopore sequencing accurately and reproducibly quantifies single-nucleotide m^6^A stoichiometry **(A)** Scatter plot comparing site-specific m^6^A stoichiometry in HEK293T cells as measured by GLORI or Nanopore direct-RNA sequencing (n = 807 sites, ≥50 reads). 1 replicate for Nanopore data. Pearson’s r = 0.96, *p* < 2e-16. Linear regression line, *y* = 0.95*x* + 0.0396. Note that the GLORI dataset^24^ only contains m^6^A data for stoichiometries > 0.05, so we filtered our Nanopore data to match. **(B)** Scatter plot comparing Nanopore site-specific m^6^A stoichiometry between wild-type and *Mettl3* KO mouse embryonic stem cells (n = 902 sites, ≥50 reads). 1 replicate each. Pearson’s r = −0.090, *p* = 0.0091. Linear regression line, *y* = −0.00208*x* + 0.00153. **(C)** Scatter plot comparing Nanopore site-specific m^6^A stoichiometry between DMSO-treated and non-targeting shRNA-treated MONOMAC-6 cells (n = 36574 sites, ≥50 reads). Each condition consists of three biological replicates merged for stoichiometry calculation. Pearson’s r = 0.99, *p* < 2e−16. Linear regression line, *y* = 1.01*x* + 0.00247.

We first confirmed that the settings used for m^6^A basecalling were optimized in our hands. We prepared our own Nanopore direct RNA sequencing dataset from HEK293T cells and compared the measured stoichiometry to the published GLORI dataset from HEK293T cells (GEO: GSE210563)^24^. Single-nucleotide resolution m^6^A calls were made from the raw Nanopore sequencing data with Dorado’s DRACH motif m^6^A model basecaller. Site-level stoichiometries were then tabulated with the Modkit pileup tool, utilizing a confidence filtering threshold of 0.99 for both modified and canonical A nucleotides; this filter ensures that only high-confidence calls are used for analysis. The m^6^A stoichiometry at each DRACH site in the transcriptome was calculated by dividing the m^6^A count by the total coverage at each site. The measured m^6^A stoichiometries using Nanopore showed a robust correlation with m^6^A stoichiometries previously measured in HEK293T cells with GLORI (Pearson’s r = 0.96, *p* < 2e-16) (Figure 1A).

It should be noted that the GLORI dataset was obtained using multiple deeply sequenced libraries (~390 million uniquely mapped reads), thus allowing this dataset to sample 176,642 m^6^A sites^24^. In contrast, our Nanopore dataset was obtained from a much lower sequencing depth (~0.5 million uniquely mapped reads) and sampled 890 m^6^A sites, of which 807 were shared with the GLORI dataset. As more Nanopore sequencing data is obtained, more m^6^A sites will have the 50 requisite reads that we used as a threshold for inclusion in our analysis of m^6^A stoichiometry, described below. However, even at this modest sequencing depth, 807 m^6^A sites could be measured with at least 50 independently sequenced reads, and in each case closely matched the stoichiometry measured by GLORI. Thus, although fewer m^6^A sites were quantified using Nanopore than GLORI, this does not indicate a weakness of Nanopore compared to GLORI, it simply reflects the sequencing depth chosen for the experiment.

To further examine the reliability of Nanopore direct RNA sequencing, we examined m^6^A stoichiometries in cells lacking METTL3, the primary methyltransferase responsible for m^6^A formation in poly(A) RNA^27,28^. We performed Nanopore direct RNA sequencing on RNA from wild-type and *Mettl3* knockout mouse embryonic stem cells (mESCs)^28^ and plotted the site-specific stoichiometries. As expected, all sites in the knockout exhibited stoichiometries of zero or near zero (Pearson’s r = −0.090, *p* = 0.0091, linear regression line, *y* = −0.00208*x* + 0.00153) (Figure 1B). These controls indicate that, in our hands and under the stated parameters, Nanopore direct RNA sequencing provides accurate m^6^A stoichiometry estimates with a low false-positive rate.

### Establishing the variability of m^6^A stoichiometry measurements between Nanopore replicates

Before we began our analysis of FTO in AML cells, we wanted to understand the variability in m^6^A measurements that can occur between biological replicates that are expected to have identical m^6^A stoichiometries at all sites. This can reveal the level of sample-to-sample variability in the measurement of m^6^A stoichiometry that should be considered experimental measurement noise. We also wanted to understand the amount of read coverage needed at any specific site to obtain accurate stoichiometry measurements. By establishing this threshold, we can reduce the likelihood that we falsely identify an m^6^A site that appears to exhibit different stoichiometries between two conditions, but is actually caused by stochastic m^6^A calls due to low sequencing depth.

For this analysis, we used two conditions that are expected to have a similar distribution of m^6^A in the transcriptome: DMSO-treated (3 replicates) and control shRNA-treated (3 replicates) MONOMAC-6 AML cells. We applied a series of thresholds from 0 to 200 for the minimum read coverage at each site and calculated m^6^A stoichiometry for all sites meeting the threshold. For each threshold, we evaluated control-to-control concordance using Pearson’s correlation coefficient of determination (R^2^), which showed a rapid increase in correlation with increasing read coverage (Figure S1A).

Next, we investigated the upper and lower limits of agreement (the range within 95% of differences are expected to fall), which showed narrower ranges at higher thresholds, indicating reduced technical variability (Figure S1B). Likewise, we examined the spread of per-site m^6^A differences (Δm^6^A) with a cumulative distribution plot, showing that most differences remained centered near zero, with progressively tighter distributions at higher thresholds (Figure S1C). However, increasing the read threshold also reduced the number of analyzable m^6^A sites (Figure S1D). Based on these analyses, for any examined m^6^A site, we selected a 50 read minimum as an optimal threshold that balances stoichiometric precision with retention of a sufficient number of analyzable m^6^A sites.

Using the 50 read coverage threshold, we examined the variability in m^6^A stoichiometry measurements by comparing each m^6^A site between the two control conditions. This scatter plot showed a highly linear correlation (Pearson’s r = 0.99, *p* < 2e−16) with a tight distribution along the *y=x* identity line (linear regression, *y* = 1.01*x* + 0.00115), and a lack of any outliers, indicating no systematic bias in m^6^A levels between the two control conditions (Figure 1C, S1E). Further, Bland-Altman analysis was performed to assess control-to-control agreement between m^6^A measurements; this method evaluates the mean difference and limits of agreement between two measurements, providing a robust estimate of reproducibility. The analysis showed that 95% of differences fell within ±0.066 of each other, with a mean difference of −0.0034, indicating close agreement between controls (Figure S1F). Based on this control-to-control variability, we defined ±0.066 as the baseline measurement variability for m^6^A stoichiometry across the transcriptome.

Overall, these experiments established a quantitative reference for detecting meaningful changes in m^6^A stoichiometry and assessing potential FTO-regulated sites.

### FTO depletion leads to marked upregulation of m^6^Am in AML snRNA

**Figure 2.**
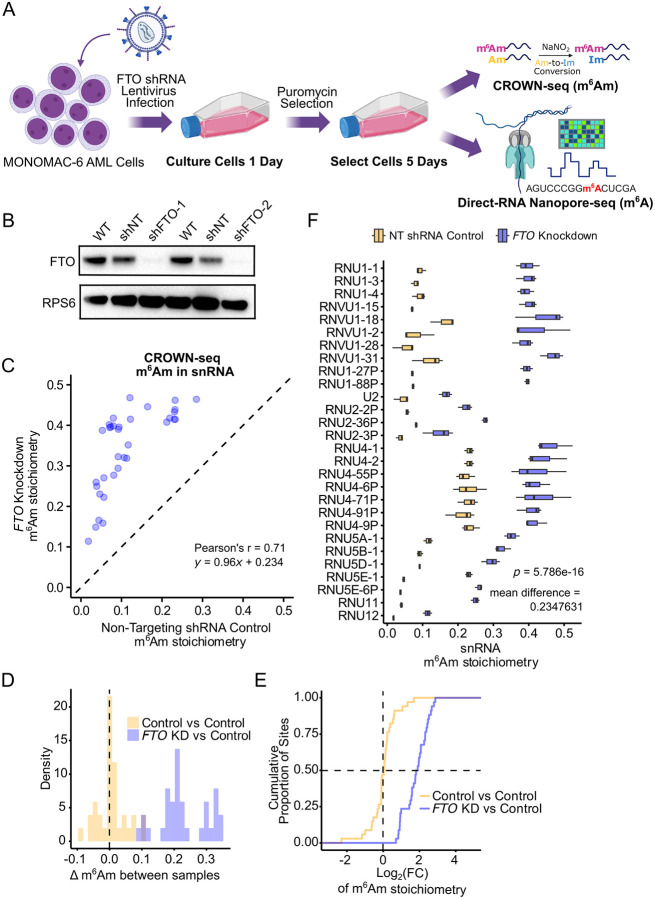
Knockdown of FTO Increases m^6^Am Stoichiometry in AML snRNAs **(A)** Schematic of lentivirus treatment and timing. Collected RNA was subjected to CROWN-seq for m^6^Am quantification, and Direct-RNA Nanopore-seq for m^6^A quantification. Created in BioRender. Jaffrey, S. (2025) https://BioRender.com/5wkk4dd. **(B)** Western blot assay confirmation of FTO knockdown by lentiviral constructs in MONOMAC-6 cells. RPS6 was used as a loading control. WT, uninfected wild-type; shNT, non-targeting shRNA control; shFTO, FTO-specific shRNA. **(C)** Scatter plot comparing snRNA Transcription Start Site-specific m^6^Am stoichiometry in Non-Targeting control vs FTO-specific shRNA infected MONOMAC-6 cells (n = 34 sites, ≥50 reads). m^6^Am stoichiometry was quantified by CROWN-seq in triplicate and averaged for plotting. Pearson’s r = 0.71, *p* = 3e−06. Linear regression line, *y* = 0.96*x* + 0.234. **(D and E)** Overlaid histogram of m^6^Am differences (Δm^6^Am) **(D)** and cumulative distribution plot of log_2_-fold change in m^6^Am stoichiometry **(E)** between comparisons: NT shRNA control replicate 1 vs replicate 2 (yellow), and *FTO* knockdown replicate 1 vs NT shRNA control replicate 1 (blue). **(F)** Boxplots of m^6^Am stoichiometry in different snRNA transcripts. Three replicates each of NT shRNA control (yellow) and *FTO* knockdown (blue) show a robust increase in m^6^Am at every measured snRNA (only transcripts with ≥50 read coverage for each replicate are included). *FTO* knockdown resulted in an average increase of 0.235 across snRNAs. Statistical significance was determined using a paired t-test on the mean stoichiometry per snRNA (n = 28 unique snRNA transcripts, *p* = 5.786e-16).

We first wanted to establish conditions to deplete FTO and to verify that FTO activity is lost in cells. To accomplish this, we tested two lentiviruses expressing different human FTO-specific shRNA by infecting MONOMAC-6 AML cells (Figure 2A). MONOMAC-6 was chosen as our primary cell line of study, as it was also used in the initial AML study showing that FTO demethylates m^6^A in mRNA^9^. We measured FTO expression by western blot after 5 days of puromycin selection (Figure 2B). Lentivirus shFTO-2 exhibited a near-complete depletion of FTO protein and was used to test the role of FTO on m^6^A levels.

We next sought to confirm that FTO depletion led to reduced FTO activity. Since a major function of FTO is to demethylate m^6^Am in the transcription start site of snRNA^6,7^, we analyzed m^6^Am levels in snRNA. As there is currently no Nanopore basecaller for m^6^Am, we used CROWN-seq^6,29^, a single-nucleotide resolution method for stoichiometric mapping of m^6^Am. CROWN-seq uses a chemical reagent that converts 2’-*O*-methyladenosine (Am) to 2’-*O*-methylinosine (Im), but leaves *N*^6^,2’-*O*-dimethyladenosine (m^6^Am) intact^6,29^. By sequencing the transcription-start nucleotide and measuring the number of adenosines (m^6^Am) and inosines (Am), CROWN-seq provides highly accurate measurements of m^6^Am stoichiometry in snRNA^6,29^.

We compared m^6^Am stoichiometry at all annotated snRNA transcription-start site positions between three replicates of control and *FTO* knockdown conditions. All measured snRNA-associated m^6^Am sites exhibited large increases in stoichiometry upon FTO depletion, with the Pearson’s correlation coefficient equal to r = 0.71 (*p* = 3e-06) and the linear regression given by *y* = 0.96*x* + 0.234 (Figure 2C). This scatter plot demonstrates a clear divergence from the *y*=*x* identity line. The baseline m^6^Am levels in snRNA are generally low^6^; however, overlaid histograms of Δm^6^Am and a cumulative distribution plot of log_2_-fold change between conditions show a clear increase in m^6^Am upon *FTO* knockdown, with most sites increasing by 2-fold (Figure 2D, E). For reference, we also show boxplots of m^6^Am stoichiometry at each snRNA transcript that could be detected (Figure 2F). The U1 snRNA gene family showed the highest increases in m^6^Am stoichiometry upon FTO depletion, with increases up to 0.347 compared to control (a 2.9-fold increase), indicating that U1 may be a mediator of FTO biology.

Overall, these data confirm that FTO is inhibited as a result of infection with the FTO shRNA-expressing lentivirus, based on both the western blot and the substantial increases in m^6^Am stoichiometry in snRNAs.

### FTO depletion does not affect the stoichiometry of m^6^A sites in poly(A) RNA

We next sought to identify the m^6^A sites in *MYC* mRNA that are demethylated by FTO in AML cells. We used the same RNA samples described above, comprising three biological replicates from the non-targeting control shRNA and three from the FTO shRNA-expressing MONOMAC-6 cells (Figure 2A). We subjected these samples to Nanopore sequencing and m^6^A basecalling. In total, we obtained 2.6 million primary mapped reads for the FTO-depleted MONOMAC-6 cells and 2.7 million reads for the control shRNA (see Table S1).

We first wanted to assess reproducibility across each replicate. To test this, we generated pairwise scatter plots between individual biological replicates, which showed high correlation and similar transcriptome-wide m^6^A stoichiometry measurements for both the control and FTO-depleted MONOMAC-6 cells (Figure S2A).

We also examined the number of m^6^A sites that reached the 50 read threshold, both globally and for *MYC* (Table S2). The number of sites increased when merging the three biological replicates. Since the sample-to-sample reproducibility was high, we used the merged dataset to maximize the number of reads at each m^6^A site, thereby bringing more sites above the 50 read threshold and achieving the highest accuracy in calculating m^6^A stoichiometry at each site for each condition.

For our analysis, we measured m^6^A stoichiometry at every DRACH site in the transcriptome. We excluded sites that had a stoichiometry of zero in both samples. This resulted in a set of 30,880 global m^6^A sites and 31 *MYC*-specific m^6^A sites, which include all m^6^A sites previously identified in *MYC* mRNA using GLORI^24^ (Table S3).

**Figure 3.**
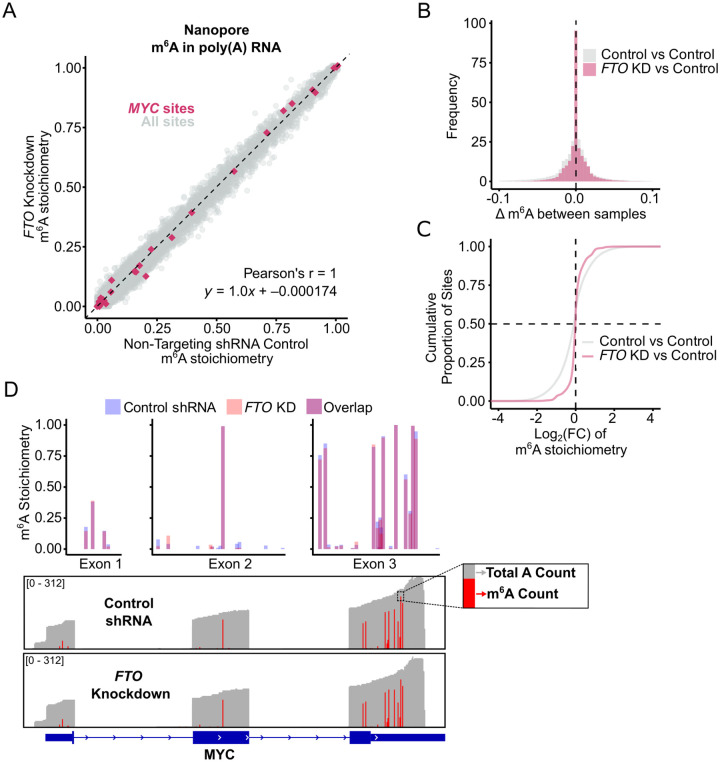
Knockdown of FTO Does Not Increase m^6^A Stoichiometry in AML mRNA **(A)** Scatter plot comparing site-specific m^6^A stoichiometry between FTO knockdown and NT-shRNA control MONOMAC-6 cells (n = 30880 sites, ≥50 reads). Each condition consists of three biological replicates merged for stoichiometry calculation. *MYC* sites are highlighted in pink (n = 31 sites). Pearson’s r = 1, *p* < 2e–16. Linear regression line, *y* = 1.0*x* + −0.000174. **(B and C)** Overlaid histogram of per-site m^6^A differences (Δm^6^A) **(B)** and Cumulative distribution plot of log_2_-fold change in m^6^A stoichiometry **(C)** between comparisons: merged DMSO control vs NT-shRNA control (grey, n = 36574 sites), and merged FTO knockdown vs NT-shRNA control (pink, n = 30880 sites). **(D)** Gene-level view of *MYC* showing consistent per-site m^6^A stoichiometry in NT-shRNA control and FTO knockdown MONOMAC-6 cells (3 replicates merged for sufficient coverage). IGV (Integrated Genome Viewer) panels (bottom) show comparable total read coverage in grey (0–312); at each DRACH site, the fraction of A’s called as m^6^A is shown in red. Overlaid m^6^A stoichiometry bar graphs (top) show no increase in m^6^A stoichiometry upon FTO knockdown.

To identify m^6^A sites that increase in stoichiometry after FTO depletion, we prepared a scatter plot comprising the calculated stoichiometry of each detected m^6^A site in the control shRNA and FTO-specific shRNA conditions (Figure 3A, S2B). Unexpectedly, we found that m^6^A stoichiometries for all DRACH sites across the AML transcriptome were essentially unchanged between conditions. The scatter plot displayed a tight correlation along the *y*=x identity line, with the Pearson’s correlation coefficient equal to r = 1.0 (*p* < 2e−16) and the linear regression line given by *y* = 1.0*x* – 0.000174. Overlaid histograms of the difference in m^6^A stoichiometry between *FTO* knockdown and control samples (Δm^6^A) and a cumulative distribution plot of log_2_-fold change in m^6^A stoichiometry confirm no apparent difference between knockdown and control (Figure 3B, C). Further, a Bland-Altman analysis showed that the m^6^A stoichiometry measurements between conditions had a mean difference of 0.00071, with 95% of site-level differences falling within ±0.031 (Figure S2C), suggesting no appreciable effect on stoichiometry beyond baseline variability (±0.066 in control comparison, see Figure S1F).

Despite observing no global increase in m^6^A stoichiometry upon FTO depletion, we considered that *MYC* may be specifically targeted. However, we again found that m^6^A stoichiometries for all DRACH sites in *MYC* (Figure 3A, highlighted in pink) were essentially identical in the FTO shRNA-expressing and control shRNA-expressing MONOMAC-6 cells. To better visualize the m^6^A sites in *MYC*, we compared IGV coverage tracks between conditions, with total read coverage shown in grey and m^6^A stoichiometry (represented as the fraction of modified reads) in red (Figure 3D). Per-site stoichiometries for control and FTO knockdown are overlaid above the IGV tracks for direct comparison. We did not identify FTO-associated changes at the surveyed *MYC* sites in our datasets.

In addition to *MYC*, other studies have suggested that FTO regulates m^6^A levels in *CEBPA*^10^, *RARA*^9,11^, *ASB2*^9,11^, and *LILRB4*^30^ mRNA. However, only *CEBPA* achieved sufficient read coverage in our data (50 reads per site) to quantify m^6^A stoichiometry. Again, we saw no difference in m^6^A stoichiometry in FTO-depleted MONOMAC-6 cells relative to the control (Figure S2D). In the case of *RARA*, *ASB2*, and *LILRB4*, none of the m^6^A sites achieved the 50 read threshold for m^6^A quantification. However, by relaxing the filter threshold to 10 reads, we could analyze three *LILRB4* m^6^A sites and ten *RARA* m^6^A sites. Even in this noisier plot, none of these sites showed increased stoichiometry in FTO-depleted cells (Figure S2E).

Overall, our quantitative mapping did not reveal the expected large increases in m^6^A stoichiometry after FTO depletion at *MYC* m^6^A sites or at other surveyed sites in the AML transcriptomes. Furthermore, our data did not show even small changes in m^6^A stoichiometry within our measurement precision.

### Pharmacologic inhibition of FTO using FB23–2 does not affect m^6^A stoichiometry in poly(A) RNA

**Figure 4.**
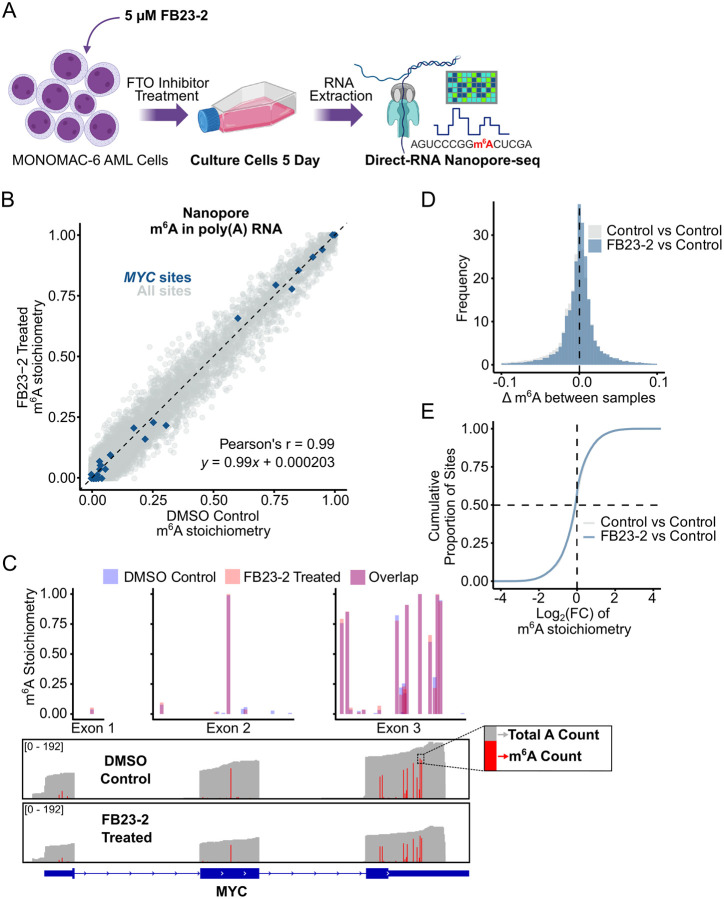
FTO Inhibition via FB23–2 Does Not Increase m^6^A Stoichiometry in AML mRNA **(A)** Schematic of FB23–2 drug treatment and timing. Collected RNA was subjected to Direct-RNA Nanopore-seq for m^6^A quantification. Created in BioRender. Jaffrey, S. (2025) https://BioRender.com/0vo3cnd. **(B)** Scatter plot comparing site-specific m^6^A stoichiometry between FB23–2-treated and DMSO-treated control MONOMAC-6 cells (n = 31324 sites, ≥50 reads). Each condition consists of three biological replicates merged for stoichiometry calculation. *MYC* sites are highlighted in blue (n = 29 sites). Pearson’s r = 0.99, *p* < 2e−16. Linear regression line, *y* = 0.99*x* + 0.000432. **(C)** Gene-level view of *MYC* showing consistent per-site m^6^A stoichiometry in DMSO-treated control and FB23–2-treated MONOMAC-6 cells (3 replicates merged for sufficient coverage). IGV panels (bottom) show total read coverage in grey (0–192), and the fraction of A’s called as m^6^A in red. Fewer reads are seen in the FB23–2-treated samples, however, stoichiometry remains unchanged. Overlaid m^6^A stoichiometry bar graphs (top) show no increase in m^6^A stoichiometry upon FTO inhibition. Note that fewer sites are displayed relative to the FTO KD experiment, as fewer sites met the 50 read coverage threshold. **(D and E)** Overlaid histogram of per-site m^6^A differences (Δm^6^A) **(D)** and cumulative distribution plot of log_2_-fold change in m^6^A stoichiometry **(E)** between comparisons: merged DMSO control vs NT-shRNA control (grey, n = 36574 sites), and merged FB23–2-treated vs DMSO-treated control (blue, n = 31324 sites). Sites with a stoichiometry of zero in both conditions were filtered out.

As an alternative approach to test the role of FTO as an m^6^A demethylase, we inhibited FTO with FB23–2, a small-molecule inhibitor reported to upregulate global m^6^A levels and induce AML differentiation and apoptosis^11^. FB23–2 was a major advance over non-selective FTO inhibitors like rhein and meclofenamic acid. Notably, FB23–2 also significantly increases m^6^Am levels at 5 μM, the concentration that is used for toxicity assays^31^. We treated MONOMAC-6 AML cells with 5 μM FB23–2 for 5 days before performing direct RNA nanopore sequencing (Figure 4A). Three biological replicates for both drug and DMSO (vehicle) conditions were subjected to Nanopore direct RNA sequencing and m^6^A basecalling. Again, the replicates showed high replicate-to-replicate similarity (Figure S3A). All replicates were merged for high-coverage single-nucleotide m^6^A stoichiometry calculation. In total, we obtained 1.7 million reads for the FB23–2-treated MONOMAC-6 cells and 2.9 million reads for the DMSO control (see Table S1).

A scatter plot of transcriptome-wide m^6^A stoichiometries (FB23–2 vs DMSO) revealed high similarity at all measured sites between the two conditions, with the Pearson’s correlation coefficient equal to r = 0.99 (*p* < 2e-16) and the linear regression line given by *y* = 0.99*x* + 0.000203 (Figure 4B, S3B). This indicates that FB23–2 treatment did not produce a global increase in poly(A) RNA m^6^A stoichiometry. Again, we highlighted *MYC* sites in the scatter plot (blue) and included a *MYC* gene-level view with IGV tracks and overlaid per-site m^6^A stoichiometries (Figure 4C). Even though the merged FB23–2 dataset showed a smaller total read coverage relative to the DMSO control, m^6^A stoichiometries remained consistent, with small differences in both directions within the bounds of normal variability (see Figure S1F). As such, we did not detect FTO-regulated *MYC* sites by FB23–2 inhibition.

To more stringently assess any shifts in global m^6^A stoichiometry after FB23–2 treatment relative to our control comparisons, we plotted histograms of the difference in m^6^A stoichiometry (Δm^6^A) (Figure 4D) and a cumulative distribution plot of log_2_-fold change in m^6^A stoichiometry between conditions (Figure 4E). Both analyses corroborated the scatter plot, demonstrating that m^6^A levels in FB23–2-treated samples do not deviate from vehicle-treated controls. We also performed a Bland-Altman analysis, which showed a mean difference between conditions of 0.00024, with 95% of site-level differences falling within ±0.055 (Figure S3C), suggesting no appreciable effect on stoichiometry beyond baseline variability (±0.066 in control comparison, see Figure S1F).

We also performed the FB23–2 treatment in MOLM-13 cells, another AML cell line used in FTO-related studies^32–34^. In this case, we only sequenced a single replicate of FB23–2 and DMSO vehicle (437,378 and 421,302 primary mapped reads, respectively) (see Table S1). As in MONOMAC-6, no global increase in m^6^A stoichiometry was detected, with a Pearson’s correlation coefficient of r = 0.99 and a linear regression line of *y* = 0.97*x* – 0.000049 (Figure S3D).

Overall, we used two approaches: pharmacologic FTO inhibition and shRNA-mediated *FTO* knockdown. Both approaches indicate that there are no detectable m^6^A sites in *MYC* or elsewhere in the transcriptome that are regulated by FTO in the tested AML cell lines.

### *FTO* knockout in HEK293T cells does not alter m^6^A stoichiometry

**Figure 5.**
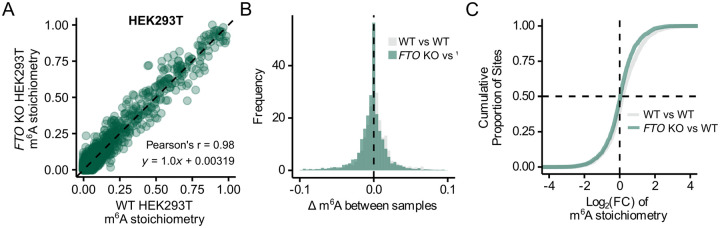
*FTO* Knockout in HEK293T Cells Does Not Increase m^6^A Stoichiometry **(A)** Scatter plot comparing site-specific m^6^A stoichiometry between *FTO* knockout and wild-type HEK293T cells (n = 4393 sites, ≥50 reads). 1 replicate each. Pearson’s r = 0.98, *p* < 2e−16. Linear regression line, *y* = 1.0*x* + 0.00319. **(B and C)** Overlaid histogram of per-site m^6^A differences (Δm^6^A) **(B)** and cumulative distribution plot of log_2_-fold change in m^6^A stoichiometry **(C)** between comparisons: wild-type HEK293T replicate 1 vs replicate 2 (grey, n = 3251 sites), and *FTO* knockout vs wild-type HEK293T control (green, n = 4393 sites).

To determine whether FTO depletion has measurable effects on m^6^A stoichiometry outside of the AML context, we analyzed HEK293T cells with complete genetic knockout of *FTO* (KO), which thus provides a definitive loss-of-function background. These cells exhibit markedly increased levels of m^6^Am in snRNA^7^. We again used Nanopore direct RNA sequencing, m^6^A basecalling, and quantified m^6^A stoichiometries at sites throughout the transcriptome. The scatter plot comparing *FTO* WT and KO samples again revealed a tight correlation along the *y=x* identity line, indicating that global m^6^A levels remain unchanged despite complete FTO loss (Figure 5A, S4A). The Pearson’s correlation coefficient was r = 0.98 (*p* < 2e-16) and the linear regression line was *y* = 1.0*x* + 0.00319, with no systematic deviation toward increased or decreased methylation across sites.

Additionally, the histogram of per-site Δm^6^A values (WT – KO) showed no global shift in either direction, with differences clustering tightly around zero (Figure 5B), and the cumulative distribution plot of log_2_-fold change in m^6^A stoichiometries between WT and KO showed no difference from that of the replicate-to-replicate WT comparison (Figure 5C). Bland-Altman analysis between WT replicates showed a mean difference of 0.0023, with 95% of site-level differences falling within ±0.058 (Figure S4B); similarly, comparison between *FTO* KO and WT samples showed a mean difference of −0.0034, with 95% of site-level differences also within ±0.058 (Figure S4C). Because both mean differences are very close to zero and the limits of agreement largely overlap, these results indicate no detectable global change in m^6^A stoichiometry following FTO knockout.

These data extend our findings to a complete *FTO* loss-of-function background and a non-leukemia cell line.

### FTO is not required for the survival of AML cell lines in culture

Based on our experiments above, FTO does not appreciably demethylate m^6^A in mRNA, at least in the tested AML cell lines and HEK293T cells. However, the FTO studies also found that FTO is essential for AML growth based on the finding that FTO depletion and inhibitors impair AML cell line growth in culture and in animal models^9–11,30^. We were surprised by this, since we did not observe a reduction in cell growth during our experiments when we used *FTO* knockdown in AML cells.

We therefore considered the possibility that the toxic effect seen with FTO shRNA in earlier studies reflected an off-target effect. Since we used different shRNA sequences than the earlier studies^30^ (see Table S4), we may not have reproduced this effect. To determine if an off-target effect could explain the reported toxicity following *FTO* knockdown in the earlier studies, we sought an independent assessment of whether FTO is required for AML cell line survival. For this, we used the Cancer Dependency Map (DepMap), a large-scale resource that was designed to identify genes that are required for cancer cell growth^35,36^. DepMap integrates both CRISPR and RNAi loss-of-function screens across >1000 cancer cell lines to quantify the degree to which each gene is needed for cell survival and proliferation^35,36^. A powerful feature of the DepMap is that it corrects for off-target effects using computational models (CERES^37^ and DEMETER2^38^). These tools deconvolute true gene dependencies from copy-number effects, off-target effects, and variable screen quality. Thus, DepMap is widely used to determine if a gene is essential for the survival of cancer or cancer subtypes like AML. We therefore asked if the DepMap Public 25Q2 dataset^39^ shows a requirement for FTO in AML cell survival.

**Figure 6.**
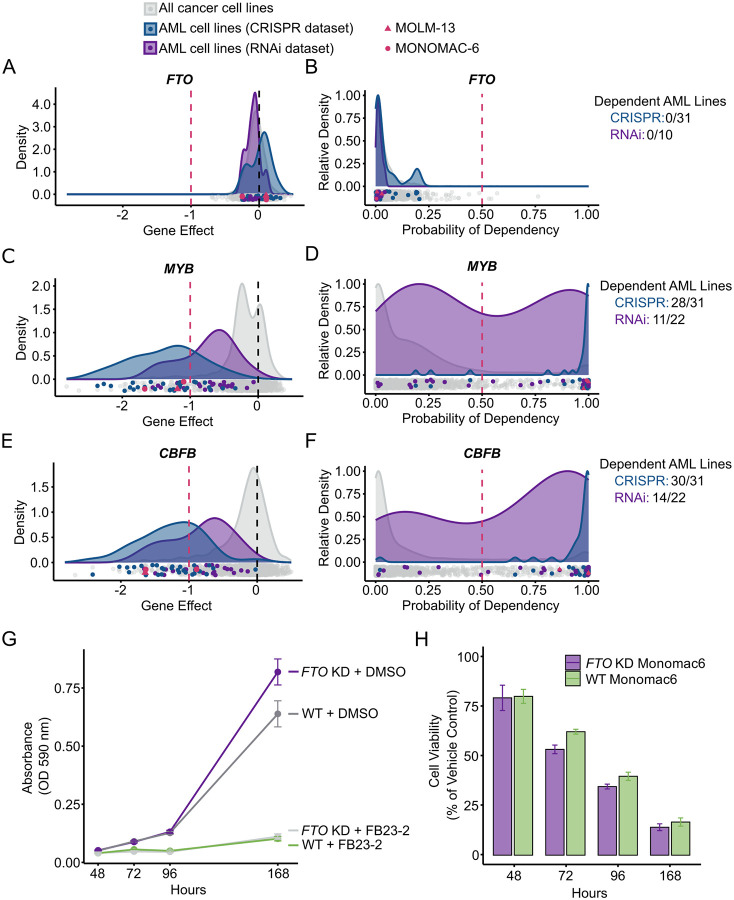
AML cells are not dependent on FTO **(A and B)** FTO is not an essential gene in most cell lines. Density plots of gene effect scores from CRISPR (blue) and RNAi (purple) datasets show values close to zero **(A)**. A lower gene effect score means a cell line is more likely to be dependent, with 0 representing a non-essential gene, and −1 representing the median of all common essential genes. All cell lines are shown below the plot (grey), with AML cell lines highlighted in blue (CRISPR screen) or purple (RNAi screen), and MONOMAC-6 and MOLM-13 called out in pink. Density plots of Probability of Dependency scores show most cell lines below the 0.5 threshold; the DepMap defines a cell line as dependent if its Probability of Dependency score is greater than 0.5. **(B)**. According to the CRISPR dataset, only 4/1183 total cell lines and 0/31 AML-specific cell lines are considered dependent on FTO; likewise, by the RNAi dataset, 0/386 total cell lines are considered dependent on FTO. **(C and D)** MYB is essential in AML cell lines. Density plots of gene effect scores show values close to −1 for highlighted AML lines **(C)**. Density plots of Probability of Dependency scores show most AML lines above the 0.5 threshold **(D)**. According to the CRISPR dataset, 168/1183 total cell lines and 28/31 AML cell lines are considered dependent on MYB; likewise, by the RNAi dataset, 23/710 total cell lines and 11/22 AML cell lines are considered dependent on MYB. **(E and F)** CBFB is essential in AML cell lines. Density plots of gene effect scores show values close to −1 for highlighted AML cell lines **(E)**. Density plots of Probability of Dependency scores show that most AML cell lines lie above the 0.5 threshold **(F)**. By the CRISPR dataset, 264/1183 total cell lines and 30/31 AML cell lines are considered dependent; by the RNAi dataset, 22/670 total cell lines and 14/22 AML cell lines are considered dependent on CBFB. **(G and H)** MTT assay cell proliferation and cell viability time courses in *FTO* WT and *FTO* knockdown MONOMAC-6 cells treated with DMSO or 5 μM FB23–2 (n = 6 replicates each). Cell proliferation (proportional to absorbance at OD=590 nm) was negatively affected by FB23–2 in both *FTO* WT and KD samples **(G)**. Cell viability, calculated as the percent absorbance in drug-treated over vehicle-treated control, showed a comparable reduction in both wild-type and FTO-depleted MONOMAC-6 cells after FB23–2 treatment **(H)**. Error bars represent mean ± standard error of the mean.

To answer this, we plotted the dependency of cell lines on FTO across all 1183 cancer cell lines using both the CRISPR and RNAi datasets^39,40^. Using both depletion approaches, only 4 out of 1183 cancer cell lines exhibited dependence on FTO in the CRISPR screen, and no cell lines were dependent in the RNAi screen. Notably, none of the 31 AML cell lines showed FTO dependency in either approach (Figure 6A, B). As controls, we also examined the dependencies of *MYB* and *CBFB*, which are known essential genes in AML^41–44^. For *MYB*, 28 out of the 31 AML cell lines were dependent in the CRISPR screen and 11 out of 22 AML cell lines were dependent in the RNAi (Figure 6C, D). Similarly, for *CBFB*, 30 out of the 31 AML cell lines were dependent based on the CRISPR screen, and 14 out of 22 AML cell lines were dependent in the RNAi screen (Figure 6E, F). These controls show that the DepMap can identify genes essential for AML survival, and FTO is not identified as such a gene.

Overall, by using DepMap, we could get an independent assessment of the role of FTO in AML cell line survival using multiple CRISPR guide RNAs and multiple shRNAs. Across AML lines, FTO gene effect score distributions centered near zero in CRISPR and RNAi datasets, and probability of dependency scores remained below 0.5, contrasting with AML-essential genes such as *MYB* and *CBFB*.

### FB23–2 cytotoxicity persists in FTO-deficient cells

Because the DepMap and our own data suggest that FTO is not needed for AML cell line survival, it was confusing to us why the FTO inhibitor FB23–2 would impair AML survival^11^. FB23–2 has been rigorously shown to bind and inhibit FTO, and has minimal effects on other dioxygenase enzymes^11^. However, we considered the possibility that FB23–2 has off-target effects that cause its cytotoxicity. To test this, we compared cytotoxicity induced by FB23–2 in FTO-deficient MONOMAC-6 cells. Surprisingly, MTT viability assays revealed FB23–2 is toxic in MONOMAC-6 cells lacking FTO (Figure 6G, H). Notably, both wild-type and FTO-deficient cells showed similar sensitivity to FB23–2.

We also evaluated HEK293T cells, which are not leukemia-derived, and tested both wild-type and *FTO* knockout (KO) lines. Again, FB23–2 treatment produced significant toxicity even in the FTO KO cells (Figure S5A, B).

Overall, our findings show that FB23–2 is cytotoxic to FTO-deficient cells, which argues against the idea that its toxicity is due to FTO inhibition. FB23–2 may still have therapeutic benefits, but its mechanism of action remains to be determined.

## DISCUSSION

A foundational concept in epitranscriptomics is that m^6^A is a reversible modification. After the initial discovery that FTO could demethylate m^6^A^3^, a series of seminal studies used transcriptome-wide mapping of m^6^A sites in control and FTO-deficient AML cell lines and revealed that FTO demethylates m^6^A in specific regions of specific transcripts, especially the *MYC* mRNA. Numerous methodologically similar studies subsequently identified FTO-targeted m^6^A sites in numerous other cancers and disease contexts^12–14^. However, these studies used non-quantitative methods for discovering m^6^A sites whose levels increase after FTO depletion. By using new methods that enable quantitative mapping of m^6^A stoichiometry in FTO-depleted MONOMAC-6, MOLM-13, and HEK293T cells, we found that FTO does not detectably demethylate any m^6^A site that could be measured, with changes in stoichiometry falling within the established baseline variability of ±0.066. Notably, this included m^6^A sites in *MYC*, the key proposed FTO target implicated in its oncogenic function^10^. Our study does not reproduce the reported m^6^A changes in the systems we tested, and it supports alternative interpretations that warrant further mechanistic work. Furthermore, our study points to the importance of re-evaluating the widespread concept that FTO is an ‘m^6^A eraser’ using new quantitative methods.

The prior studies identified FTO-regulated m^6^A sites by searching for “m^6^A peaks” that increased in height after FTO depletion^9–11^. These peaks were interpreted as reflecting sites with increased m^6^A abundance. These conclusions were based on MeRIP-seq, which uses m^6^A antibodies to immunoprecipitate m^6^A-containing RNA fragments for sequencing^15^. However, because MeRIP-seq peaks exhibit considerable variability in heights, even when comparing biological replicates^17^, a change in peak height after FTO depletion could simply be the normal peak height variability. Thus, MeRIP-seq is not appropriate for discovering differential m^6^A modifications between samples. This issue of poor quantitative accuracy is a general feature of antibody-based methods, e.g., ChIP-seq, which also has false positives and poor quantitative accuracy^45^. For this reason, modifications to MeRIP-seq have been described that markedly increase its accuracy (m^6^A-seq2)^46^. To the best of our knowledge, only MeRIP-seq, but not m^6^A-seq2 or other quantitative methods, has been used when identifying differential m^6^A sites in studies of FTO.

Because MeRIP-seq is not intended for quantitative measurements of m^6^A, if any m^6^A site is found to be differentially regulated based on MeRIP-seq, it should be biochemically validated. Several methods exist for quantifying m^6^A stoichiometry at specific nucleotide positions in the transcriptome. One such method is SCARLET, which has been used to measure m^6^A stoichiometry at numerous sites using site-specific cleavage and radiolabeling^47^. Other quantitative m^6^A mapping methods have also been developed^48–52^, although we are unaware of these methods being used in studies of FTO. We suggest that m^6^A sites should only be defined as “FTO regulated” if the exact m^6^A stoichiometry before and after FTO depletion is reported and shows clear increases.

With the recent improvements in Nanopore sequencing, transcriptome-wide m^6^A stoichiometry measurements are markedly simplified, allowing us to measure m^6^A stoichiometries before and after FTO depletion. In Nanopore direct RNA sequencing, m^6^A nucleotides in the nanopore produce specific electric signals that allow for highly accurate detection of m^6^A in single strands of native mRNA. The accuracy of Nanopore sequencing is highly dependent on the settings used to interpret the data^20,22,23,25^, but with stringent modification probability filtering, the accuracy is quite high and matches the current state-of-the-art method, GLORI, a chemical conversion method^24^. We confirmed that Nanopore sequencing of HEK293T cell poly(A) RNA produces m^6^A stoichiometries that closely match the results of GLORI. Notably, among the 807 m^6^A sites that we analyzed, every one of them closely matched the stoichiometry measured by GLORI. Thus, a false-negative, i.e., a site that has high stoichiometry but is measured as low stoichiometry, seems unlikely. Overall, Nanopore direct RNA sequencing provides a simple and efficient method for accurately assessing the methylation state of any adenosine in the transcriptome.

We chose to use Nanopore rather than GLORI because Nanopore enables analysis of m^6^A in full-length transcripts. Long-read sequencing is particularly valuable when studying FTO, as FTO depletion can affect splicing and poly(A) site selection^53^. Since splicing patterns influence m^6^A levels^54–56^, different spliced or processed isoforms can have different m^6^A stoichiometries. Therefore, an apparent change in m^6^A stoichiometry following FTO depletion, as detected by GLORI, could in fact reflect shifts in isoform abundance or RNA processing, rather than true changes in methylation. In contrast, Nanopore sequencing allows stoichiometry analysis at the isoform level, thereby helping to disambiguate methylation from isoform effects. While we did not observe significant FTO-dependent differences in m^6^A stoichiometry, this approach remains valuable for future studies in which such changes might occur. In those scenarios, isoform-resolved analysis could validate genuine FTO-mediated demethylation events.

The idea that FTO is an m^6^A demethylase should be easily demonstrated by depleting FTO and observing increases in m^6^A stoichiometry at the FTO-targeted sites. Although we did not test every AML cell line used in earlier studies, we focused on MONOMAC-6 since it was previously used to show that FTO regulates m^6^A^9–11,30^. The lack of effect of FTO in regulating m^6^A in our MONOMAC-6 data is so inconsistent with the prior studies that it calls into question whether FTO has a physiologically relevant ability to demethylate m^6^A. Nevertheless, certain caveats about our study should be noted:

First, we cannot detect m^6^A in rare transcripts since we require a minimum read depth to accurately quantify m^6^A stoichiometry. In the MONOMAC-6 experiments, we were able to survey 2,881 genes (30,880 m^6^A sites) with a 50 read sequencing depth. Increased coverage was obtained when reducing the threshold to 25 reads (4,931 genes, 52,157 m^6^A sites), which similarly showed no clear evidence of sites that exhibit increased stoichiometry after FTO depletion. We chose the 50 read threshold since it exhibits lower statistical noise and did not compromise our ability to assess m^6^A sites in *MYC*, the main focus of our study. To survey more m^6^A sites, more read depth could be easily obtained. However, our inability to detect any clearly FTO-regulated site so far suggests that FTO-regulated m^6^A sites, if they exist at all, are rare.

A second limitation with nanopore sequencing is that sequencing accuracy is reduced at 5’ ends, preventing m^6^A identification in this region^57^. Newer 5’ ligation methods may help to resolve this problem^58^. However, this limitation does not affect our studies since the putative FTO-regulated m^6^A sites reported in *MYC* were not in the mRNA 5’ ends.

Third, although the original studies reported large increases in m^6^A peaks after FTO depletion, it is possible that these peak differences represent subtle changes in very low stoichiometry m^6^A sites, e.g., a change from 1% to 3% stoichiometry. This is a sizeable fold-increase in m^6^A, but this very small absolute difference may be difficult to detect by Nanopore. However, it is unlikely that such a small stoichiometry change would meaningfully alter the *MYC* mRNA. This issue highlights the importance of measuring the absolute stoichiometry of m^6^A sites rather than fold-change in peak heights.

Fourth, prior studies^9–11,30^ reported increased m^6^A levels in poly(A) RNA after FTO depletion based on dot blots and mass spectrometry, supporting a role for FTO in m^6^A demethylation. However, recent work has questioned the validity of inferring m^6^A stoichiometry from mass spectrometry^59^. For example, in bladder cancer, mass spectrometry indicated reduced m^6^A, but GLORI revealed no global change in m^6^A stoichiometry. Instead, shifts in transcript abundance explained the discrepancy: bladder tumors upregulated mRNAs with inherently low m^6^A, diluting the overall m^6^A/A ratio measured by mass spectrometry. This highlights that mass spectrometry reflects transcript composition rather than site-specific methylation. Since FTO depletion induces cellular differentiation^9,11,30^, changes in m^6^A levels detected by bulk methods may arise from altered gene expression, not direct demethylation. In contrast, site-specific m^6^A mapping shows that FTO does not measurably target m^6^A.

Another concern with mass spectrometry measurements of m^6^A measurements, especially after knockdown or overexpression of FTO, is that FTO levels can influence 3’UTR lengths^53^. FTO depletion leads to shortened 3’UTRs since FTO depletion triggers use of proximal polyadenylation sites^53^. Since the distal 3’UTRs are relatively low in m^6^A, global shortening or increasing 3’UTR lengths can markedly influence m^6^A/A measurements in mass spectrometry. When these 3’UTR regions are shortened after FTO depletion, m^6^A/A ratios would be expected to increase, while overexpression of FTO may decrease m^6^A/A ratios. Thus, the known effects of FTO on 3’UTRs could explain mass spectrometry findings of altered m^6^A/A levels. These issues highlight the importance of directly measuring m^6^A stoichiometries at specific sites rather than relying on global measurements of m^6^A/A ratios.

Fifth, it is possible that m^6^A levels increase following FTO inhibition, but the affected mRNAs are degraded too rapidly to be detected. While this remains a possibility, the published studies did not report this effect. Instead, they found increased m^6^A levels after FTO inhibition, which should have been readily detectable by us using Nanopore sequencing.

If FTO does not demethylate m^6^A, what is its function? We have shown that m^6^Am is much more efficiently demethylated by FTO^4^, and *FTO* knockout tissues show markedly increased m^6^Am in snRNA^6,7^. It has been argued by others that both m^6^A and m^6^Am are targeted by FTO, but FTO’s demethylation of m^6^A mediates its biologically relevant effects, especially in AML, since FTO directly targets key oncogenic drivers like *MYC*^9,10,60^. In light of the re-analysis presented here, which shows a lack of change in m^6^A but a clear increase in m^6^Am stoichiometry in snRNA after FTO depletion in AML cells, it seems likely that the effects of FTO depletion are due to elevated snRNA m^6^Am, rather than elevated m^6^A.

Although the main purpose of our study was to evaluate the demethylase function of FTO, our study also identified some other issues that were unexpected based on the published literature. One of the most confusing aspects of our study is that we saw no effects of FTO depletion on cell viability. In the earlier studies, FTO was described as an essential gene, specifically for AML cells, and its depletion led to cell death in several contexts, including cell culture^9–11,30^. Some more recent published studies have also reported that FTO depletion leads to impaired growth in various leukemia cell lines and in certain hematologic cell types in mice^61,62^. However, other studies have found no such effects in cultured leukemia cell lines. For example, *FTO* knockout K562 leukemia cells exhibited normal growth^31^, and *FTO* knockdown in a variety of leukemia cell lines had no effect on growth, although it did affect cell growth after kinase inhibitor treatment^63^.

We therefore turned to the DepMap, which is particularly powerful for determining essential genes because it uses multiple guide RNAs and shRNAs. DepMap reports that FTO depletion does not affect cell viability. It is therefore not clear why the DepMap and our work would show no cell growth defects. In some cases, DepMap fails to identify genes that are known to be essential for leukemia survival, such as DNMT3A or histone deacetylases; however, these false negatives are due to redundant paralogs that prevent cellular effects when only one paralog is deleted. No known FTO paralog exists, and FTO depletion on its own was reported to impact leukemia cell growth^9–11,30^. Thus, an FTO paralog is unlikely to explain why FTO is not detected as a leukemia dependency gene in DepMap. Instead, it is possible that some subtle difference in culturing conditions accounts for these differences. Regardless, FTO is not absolutely required for survival. Instead, the role of FTO depends on some subtle feature that is not readily reproduced.

We also cannot rule out a potential non-specific toxic effect of the shRNA used in the prior studies. shRNA can have non-specific knockdown effects on other targets, as has been widely reported^64^. Thus, an off-target effect of the FTO shRNA might account for the observed toxicity rather than FTO depletion.

Since we find that FTO is dispensable for AML cell line survival, it was not clear to us why the FTO inhibitor FB23–2 would have anti-leukemic effects^11^. In our hands, FB23–2 was toxic to AML and HEK293T cells lacking FTO. Thus, FB23–2 inhibits something other than FTO to cause cell death in these cells. While this study was in preparation, another group similarly reported that FB23–2 mediates its toxicity in an FTO-independent manner and showed that it targets DHODH, a key enzyme in uridine biosynthesis^31^. Since there is considerable interest in developing new FTO inhibitors along with “degraders” based on FB23–2^34^, we suggest that FTO inhibitors be routinely screened in *FTO* knockout cell lines to ensure that cytotoxic effects are indeed mediated by FTO inhibition.

## STAR Methods

### EXPERIMENTAL MODEL AND SUBJECT DETAILS

#### Cell lines

HEK293T (wild-type and *FTO* knockout cells, previously described by Mauer et al.^7^) were cultured in DMEM (Gibco, #11995065). MOLM-13 were grown in RPMI-1640 (ThermoFisher, 22400105). MONOMAC-6 were grown in RPMI-1640 supplemented with 2 mM L-Glutamine (ThermoFisher, 25030081), MEM NEAA (Life Technologies, 11140050), 1 mM sodium pyruvate (Life Technologies, 11360070), and 10 μg/ml human insulin (Life Technologies, 12585014). All media were supplemented with 10% fetal bovine serum (FBS) and 1X penicillin-streptomycin (Gibco, #15140148).

*Mettl3* KO and WT mESCs were previously described by Geula et al.^28^ and were a gift from S. Geula and J.H. Hanna (Weizmann Institute of Science). All mESCs were grown in gelatinized (0.1% gelatin in water, EmbryoMax ES-006-B) tissue culture plates in mESC media (KnockOut DMEM (Gibco #10829018), 15% heat-inactivated fetal bovine serum (FBS) (Gibco #26140079), 100 U/ml penicillin, 100 μg/ml streptomycin (Gibco #15140122), 1× GlutaMax (Gibco #35050061), 55 μM β-mercaptoethanol (Gibco #21985023), 1× MEM non-essential amino acids (Gibco #11140076), 1,000 U/ml LIF (Millipore ESG1107), 3 μM CHIR99201 (Sigma Aldrich SML1046), 1 μM PD0325901 (APExBIO A3013)).

Cells were incubated at 37 °C in a humidified atmosphere containing 5% CO_2_ and passaged as needed using TrypLE Express (ThermoFisher, 12604039).

#### Lentiviral constructs

pLKO lentiviral shRNA constructs and Non-Target control shRNA constructs were purchased from the Gene Editing & Screening (GES) Core Facility at MSKCC. Human FTO shRNA 1 cloneId: TRCN0000246247. Human FTO shRNA 2 cloneId: TRCN0000257473. Non-targeting control shRNA: TRC2 pLKO.5-puro Non-Target shRNA Control. Packaging plasmid: psPAX2(gag/pol/rev/tat). Envelope plasmid: pMD2.G (VSV-G).

### METHODS DETAILS

#### Lentiviral shRNA Knockdown

pLKO lentiviral shRNA constructs and Non-Target control shRNA constructs were purchased from the Gene Editing & Screening (GES) Core Facility at MSKCC (See Table S4). For lentiviral packaging, HEK293T cells were seeded in 100mm plates and allowed to reach 50–80% confluency. 2.5 μg of Lentiviral shRNA vectors were co-transfected with packaging plasmids (psPAX2 and pMD2.G) to each plate using FuGENE HD transfection reagent (Promega, E2311) according to the manufacturer’s instructions. Viral particles were harvested 24 hours, 48 hours, and 72 hours post-transfection, filtered through a 0.45 μm filter, pooled, and concentrated with Peg-it Virus Precipitation Solution (System Bioscience, #LV810A-1). 2×10^6^ MONOMAC-6 and MOLM-13 cells were suspended in a 12-well plate in 1 mL RPMI media containing 8 μg/ml polybrene (Santa Cruz Biotechnology, SC-134220). For each shRNA, a titration of virus (18μl:6μl:2μl) was used for infection. 48 hours post-infection, stable knockdown cells were selected with 1 μg/ml puromycin (Invivogen, ANT-PR-1) and cultured for an additional 5 days before collection in TRIzol LS. Given that each titration volume showed similar results, cells infected with 18 μl lentivirus were chosen for RNA and protein collection.

#### FB23–2 drug treatment

MONOMAC-6 and MOLM-13 cells were seeded in triplicate at 0.5×10^6^ cells/ml in 6-well plates. Cells were treated with 5 μM FB23–2 (MedChem Express LLC, #HY-127103) or 0.05% DMSO vehicle control and cultured for 5 days before collecting RNA in TRIzol LS.

#### RNA extraction and mRNA purification

Cellular total RNA in TRIzol LS (ThermoFisher #10296028) was extracted by Direct-zol RNA Miniprep kit (Zymo #R2070) or by Phenol Chloroform extraction. mRNA was purified by Dynabeads Oligo (dT)25 (Ambion #61002).

#### Cell Viability Assay

The effect of FB23–2 on cell viability was determined by 3-(4,5-dimethylthiazol-2-yl)-2,5-diphenyl tetrazolium bromide (MTT) assay. MONOMAC-6 (wild-type and *FTO* knockdown) cells were plated in 5 round-bottom 96-well plates with 5×10^4^ cells per well in 5 μM FB23–2 or 0.05% DMSO vehicle control and 6 replicates per condition. MTT assay was performed following Abcam’s instructions, with 5 timepoints: 24 h, 48 h, 72 h, 96 h, and 168 h. On each day, one plate was sacrificed by spinning down at 1,000 × g for 5 min at 4°C, followed by aspiration and resuspension in 50 μl serum-free RPMI media and 50 μl of 5 mg/ml MTT solution (Abcam, ab146345–500mg) in PBS. The plate was placed back in the incubator for 3 hours before adding 150 μl of MTT solvent (4 mM HCl, 0.1% NP40 in isopropanol) to dissolve formazan crystals by shaking on an orbital shaker for 15 min. Absorbance was read at OD = 590 nm, and DMSO vehicle-treated wells were considered as controls. Cell viability was calculated as:

$$\text{Cell Viability}\left(\%\right)=\left(\text{Mean absorbance of FB}23-2\;\text{treated group}/\text{Mean absorbance of control group}\right)\times 100$$

#### Western Blotting

Cells were lysed in lysis buffer [25 mM HEPES, pH 7.6, with 1.5 mM MgCl_2_, 420 mM NaCl, 0.5% NP-40, 0.2 mM EDTA, 25% (v/v) glycerol, 1 mM DTT, 1x protease inhibitor cocktail (ThermoFisher, 1861279)] with agitation for 30 min at 4°C. Supernatant was collected by spinning at 4°C at 16,000 × g for 20 min and quantified by Bradford assay (Bio-Rad, 5000006). 10 μg cell lysate was run on each lane of a NuPage 4–12% Bis-Tris pre-cast gel (ThermoFisher, NP0323BOX) and then transferred to a PVDF membrane (MilliporeSigma, IPVH00010). The membrane was blocked with 5% milk and blotted RAovernight at 4°C with 1:1000 anti-FTO rabbit monoclonal antibody (Abcam, ab124892) or 1:2500 anti-RPS6 rabbit monoclonal antibody (Cell Signaling, 2217T) in 5% BSA/ TBST/ 0.1% NaN3 solution. 1:5000 HRP-conjugated anti-rabbit IgG antibody (Cytiva, NA934) was used as a secondary antibody in 5% milk for 1 h at room temperature. Immunoblots were developed using Amersham ECL Western Blotting Detection Reagent (Cytiva, RPN2209) on a Bio-Rad ChemiDoc Imaging system.

#### Nanopore direct-RNA library preparation and sequencing

RNA sequencing libraries were prepared from total RNA or mRNA using the Nanopore Direct RNA Sequencing kit (ONT, SQK-RNA004) according to the manufacturer’s instructions, with some notable adjustments. Samples were multiplexed following the SeqTagger method from Pryszcz et al. 2025^23^ using the custom RT adapters for the b04_RNA004 model (see Table S4). In brief, 500 ng total RNA or 100 ng polyA-selected RNA in 8 μl nuclease-free water were barcoded with 1μl custom RT adapters via ligation with 1.5 μl T4 DNA ligase (NEB, M0202L), 3 μl 5x NEBNext Quick Ligation reaction buffer (NEB, B6058S), 0.5 μl diluted RCS, and 1 μl SUPERase·In RNase Inhibitor (ThermoFisher #AM2696) for 15 min at room temperature. Samples were then reverse transcribed with 1 μl Induro Reverse Transcriptase (NEB, M0681L), 8 μl 5x Induro buffer, 2 μl 10 mM dNTP, and 14 μl nuclease-free water following the incubation protocol: 55°C for 50 min, 70°C for 10 min, and 12°C hold. RNA-cDNA products were cleaned up with 1.8X AMPure XP Beads (Beckman, A63881) and washed twice in 70% ethanol before eluting in water and pooling the 4 barcoded libraries together for a total of 20 μl. Next, the pooled library was ligated with 6 μl RNA ligation adapter (RLA), 3 μl T4 DNA ligase, 8 μl 5X NEBNext Quick Ligation buffer, and 3 μl nuclease-free water, incubating for 10 min at room temperature. The ligation product was cleaned up with 0.8X AMPure XP beads, washed twice with the provided wash buffer (WSB), and eluted in 33 μl RNA elution buffer (REB). Each library was quantified using 1 μl for Qubit 1x dsDNA HS assay (ThermoFisher, Q33231), with a recovery aim greater than 30ng total. Samples were loaded onto primed MinION (ONT, FLO-MIN004RA) or PromethION flow cells (ONT, FLO-PRO004RA) and sequenced on a MinION or PromethION for 2–22 hours, acquiring 150 Mb to 7.6 Gb (see Table S1 for details).

#### Flow Cell Nuclease Flushing

For Flow Cells which were washed and reused, a modified Flow Cell wash mix was prepared^23^ with 20 μl TURBO DNase (2U/μl) (Thermo Fisher, AM2238) and 380 μl DIL (wash diluent, provided with ONT EXP-WSH004). Wash mix was loaded onto Flow Cell and incubated for 20 min before storing or loading another library.

#### Nanopore Data Analysis

The raw POD5 sequencing data files were basecalled using Dorado v1.0.0 with the rna004_130bps_sup@v5.2.0 m6A_DRACH modification model (for supplementary analysis, the rna004_130bps_sup@v5.1.0 inosine_m6A model was used, with m^6^A calls extracted). The raw POD5 files were also used to generate a demultiplexed pod5.demux.tsv.gz file following the SeqTagger protocol with model b04_RNA004^23^. Basecalled BAM files were mapped to the human genome hg38 (or mouse genome mm10) using the Dorado aligner with Minimap2^65^ options “-ax splice -k 14”. Aligned reads were demultiplexed with the SeqTagger bam_split_by_barcode.py script using the previously generated pod5.demux.tsv.gz file^23^. The demultiplexed reads were sorted and indexed with SAMtools^66^ v1.2.20 and visualized using IGV^67^ v2.18.2 (a base modification likelihood threshold of 0.99 was used for visualizing m^6^A calls). For increased read coverage, replicates were merged with SAMtools merge before being sorted and indexed. Modification bed tables (mod.bed) were compiled using Modkit v0.2.8 with a pileup filter threshold of 0.99 for all bases. Mod.bed files were loaded into RStudio v2024.12.1+563 for subsequent analysis and figure drawing by a custom script.

#### snRNA CROWN-seq library preparation and sequencing

To study snRNA m^6^Am stoichiometries, we used a modified protocol of CROWN-seq^6^, in which 2’-*O*-methyladenosine (Am) is chemically converted into 2’*O*-methylinosine (Im) and *N*^6^,2’*O*-dimethyladenosine (m^6^Am) remains unconverted, allowing for m^6^Am quantification. Short RNAs (17–200 nt) were isolated from total RNA using RNA Clean & Concentrator-5 (Zymo Research, R1016), following the Purification of Small and Large RNAs into Separate Fractions protocol. 100 ng of the small RNA fraction, in 8 μl nuclease-free water, was used as input. Following the conditions from the GLORI2.0 protocol^68^, samples were converted in deamination buffer (13μl glyoxal, 10μl DMSO, 10μl 5 M NaNO_2_, 5μl H_3_BO_3_, 4μl 500 mM MES (pH 6.0)) for 10 min at 50°C. Next, samples were ethanol precipitated, and the glyoxal adduct was removed with deprotection buffer (500 mM TEAA pH=8.6, 47.5% deionized formamide, 2mM EDTA) for 10 min at 95°C. RNA was collected again by ethanol precipitation, denatured for 2 min at 65°C, and spiked with 10 ng of Drosophila melanogaster embryo Poly A+ RNA (Takara Bio, #636224) for QC. To eliminate contamination of RNA species with a 5’-triphosphate or 5’-monophosphate, a dephosphorylation reaction was set up with 5 μl 10X CutSmart buffer, 5 μl Quick CIP (5 U/μl) (NEB, M0525L), and 1 μl SUPERase·In RNase inhibitor (ThermoFisher #AM2696) for 2 hours at 37 °C. The reaction was cleaned up using a Zymo RCC-5 column, and the RNA was eluted in 15.5 μl water. Next, RNA was denatured for 5 min at 65°C before removing the m^7^G-cap with 2 μl mRNA Decapping Enzyme (100U/μl) (NEB, M0608S), 2 μl 10X Decapping Enzyme Reaction Buffer, and 0.5 μl SUPERase·In RNase inhibitor for 1 hour at 37°C. The decapped 5’-monophosphate RNA was purified with ethanol-AMPure XP (RNA:beads:ethanol=1:2:3), washed twice in 80% ethanol, and eluted in 11.5 μl water. 5’ ligation was carried out with 20 pmol of 5’ adapter (see Table S4), 2 μl T4 RNA ligase 1 (ssRNA Ligase, 30U/μl) (NEB, M0437M), 4 μl 10X T4 RNA Ligase Buffer, 4 μl 10mM ATP, 0.5 μl SUPERase·In RNase inhibitor, and 16 μl 50% PEG8000 for 2 hours at 25 °C. 5’ ligation product was again purified with ethanol-AMPure XP and eluted in 16 μl water. End repair was carried out with 1 μl T4 PNK (NEB, M0201L), 2 μl 10X T4 PNK Reaction Buffer, and 1 μl SUPERase·In RNase inhibitor for 30 min at 37 °C. End-repaired RNA product was purified with ethanol-AMPure XP and eluted in 12 μl water. 3’ adapter incorporation and cDNA synthesis were carried out via a template switching reaction with an R2R RNA/R2R DNA heteroduplex adapter based on TGIRT-seq^69–71^ (since A is assumed to be converted from GLORI, the R2R DNA mix consists of A, G, or C overhangs in a 1:1:2 ratio, see Table S4). The template switching reaction was first pre-incubated at room temperature for 30 min with 2 μl 10X annealed R2 RNA/R2R DNA heteroduplex mix (100 nM final conc.), 1 μl Induro RT (200U/μl) (NEB, M0681L), 4 μl 5X Induro RT Buffer, and 1 μl SUPERase·In RNase inhibitor; next, 1 μl 10mM dNTP was added before incubating for 5 min at 25 °C, 15 min at 55 °C, 1 min at 95 °C, and holding at 4 °C. 5 μl of the cDNA product was carried forward for indexing PCR, mixed with 15 μl water, 25 μl Phusion High-Fidelity PCR Master Mix with HF Buffer (NEB, M0531L), 2.5 μl i5 indexing primer, and 2.5 μl i7 indexing primer (NEB, E7780S). Cycling conditions were 98 °C for 30 sec, then 14 cycles of 98 °C 15 seconds, 65 °C 30 seconds, and 72 °C 15 seconds, with a final 5 min 72 °C extension. Two rounds of 0.9X AMPureXP bead purifications were performed to remove primers. 10–20 ng indexed library was obtained for each library. The libraries were mixed and sequenced by NovaSeqX.

#### snRNA CROWN-seq data processing

The read pairs were first quality trimmed by Cutadapt v3.4^72^: -m 32 -q 20 -e 0.25 -a NAGATCGGAAGAGCACACGTC -A TAAN{11GATCGGAAGAGCGTCGTG. The subsequent alignment and statistics were identical to the CROWN-seq mapping pipeline as previously described^6^. In brief, the UMIs (the 11 nt at the beginning of read1) were extracted by UMI-tools v1.1.1^73^. Next, *in silico* converted read pairs (read1 A-to-G, read2 T-to-C) were aligned by HISAT2^74^ against the respective converted reference genome and transcriptome. Only read1 reads without 5’ end softclips were used for 5’ end analysis. Pileup was performed to obtain the read coverages of every 5’ end in the transcriptome. Non-conversion rates of the transcription start nucleotides were calculated by A counts over A and G counts. Only canonical snRNA TSSs based on annotations from Gencode v45 were considered for our analysis.

#### DepMap Analysis

Gene effect scores were obtained from both the DepMap CRISPR (DepMap Public 25Q2+Score, Chronos) and RNAi (Achilles+DRIVE+Marcotte, DEMETER2) datasets^39^. CRISPR gene dependency probability scores were obtained from the DepMap Public 25Q2 dataset, and RNAi gene dependency probability scores were obtained from the 2019 DEMETER 2 Combined RNAi dataset^40^. We extracted the gene dependency probability scores of 1,183 cell lines for the genes *FTO, MYB*, and *CBFB* and plotted them to identify dependent cell lines. Gene dependency probability scores greater than 0.5 indicate that a cell line is dependent on the gene.

#### Genomic assembly and annotations

The following annotations and genomic sequences were used in this study: human, Gencode v45 (GRCh38); mouse, Gencode M10 (GRCm38.p4)

### QUANTIFICATION AND STATISTICAL ANALYSIS

Statistical analysis for CROWN-seq was performed with Python (version 3.8.7). All other statistical analyses and visualizations were performed in RStudio Server (Posit Software, version 2024.12.1) running R version 4.3.3 (2024-02-29)^75^. Correlations between m^6^A stoichiometry values were assessed using Pearson’s product–moment correlation (cor.test function in R), with both the correlation coefficient (r) and *p* value reported. For analysis of read depth thresholding, the coefficient of determination (R^2^) is reported to summarize the proportion of variance explained by the correlation. Linear regression models were fitted with the lm function to calculate regression slope and intercept values. Bland–Altman analysis was performed to assess agreement between samples, with the mean difference and 95% limits of agreement (mean difference ± 1.96 × standard deviation of differences) reported. Unless otherwise stated, statistical tests were two-sided and considered significant at *p* < 0.05. Exact r, *p*, slope, intercept, and Bland-Altman values are provided in the figure legends.

Versions of key Python packages: numpy (1.23.5); pandas (1.5.2); scipy (1.9.3); matplotlib (3.6.2); seaborn (0.12.1); matplotlib-venn (0.11.9). Versions of key R packages: ggplot2 (3.5.1); tidyr (1.3.1); dplyr (1.1.4); scales (1.3.0); patchwork (1.3.1)

### DATA AND CODE AVAILABILITY

All sequencing data can be accessed from NCBI Gene Expression Omnibus (GEO) under accession code GSEXXXXXX. The public GLORI dataset was downloaded from GEO under accession code GSE210563. The public 25Q2 DepMap dataset is available from the Broad Institute’s DepMap Consortium and is accessible at https://depmap.org/portal/download/. Original western blot images have been deposited in Mendeley Data under DOI: 10.17632/6rp3hmx2x5.1. Raw direct RNA Nanopore data (POD5 files) are available upon request. Code for statistical analyses and illustrations is available upon request.

### MATERIALS AVAILABILITY

Reagents and materials in this study are available upon request.

## Supplementary Material

Supplement 1 Table S1. Nanopore sequencing information. Related to Figures 1, 3, 4, and 5.

Supplement 2 Table S2. Total DRACH and m^6^A sites per sample. Related to Figures 1, 3, 4, and 5.

Supplement 3 Table S3. *MYC* m^6^A sites in nanopore samples compared to GLORI. Related to Figures 3 and 4.

Supplement 4 Table S4. shRNA constructs and sequencing adapters. Related to Figures 1, 2, 3, 4, 5, and 6.

1
