# Supplementary material for: FTO depletion does not alter m6A stoichiometry in AML mRNA: a reassessment using direct RNA nanopore sequencing
Source: bioRxiv. 2025 Oct 23:2025.10.22.681652. Preprint. [Version 1] doi: 10.1101/2025.10.22.681652 (PMC12633362; doi:10.1101/2025.10.22.681652)
Supplement: 1 [file NIHPP2025.10.22.681652V1-supplement-1.pdf]

bioRxiv preprint doi: https://doi.org/10.1101/2025.10.22.681652; this version posted October 23, 2025. The copyright holder for this preprint (which was not certified by peer review) is the author/funder, who has granted bioRxiv a license to display the preprint in perpetuity. It is made available under aCC-BY 4.0 International license.

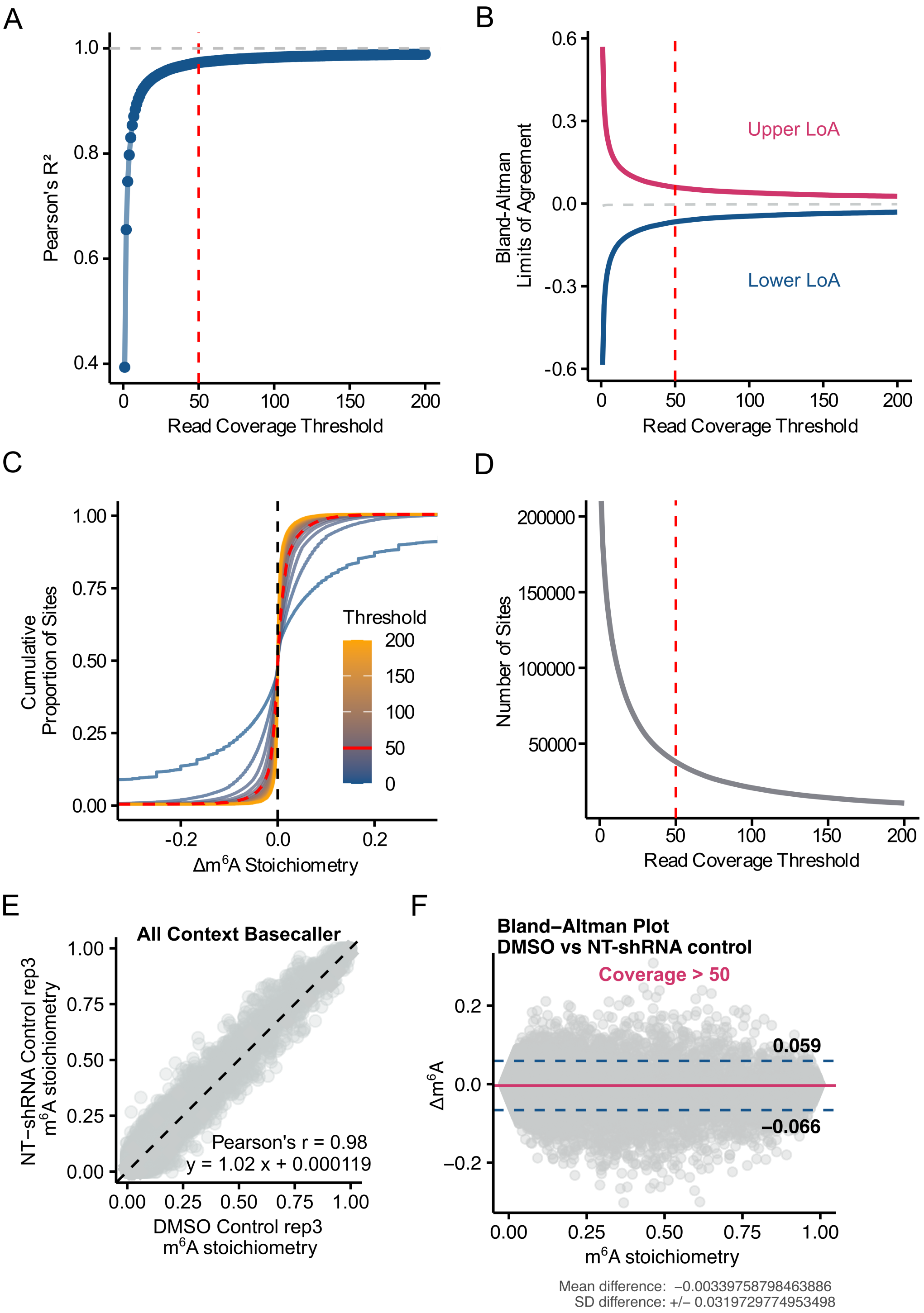

bioRxiv preprint doi: https://doi.org/10.1101/2025.10.22.681652; this version posted October 23, 2025. The copyright holder for this preprint (which was not certified by peer review) is the author/funder, who has granted bioRxiv a license to display the preprint in perpetuity. It is made available under aCC-BY 4.0 International license.

Figure S1. Justification of read depth threshold for m^{6}A stoichiometry analysis. Related to Figure 1.

(A) Pearson's correlation (R^2) between m^{6}A stoichiometry values for DMSO control (3 replicates, merged) and NT-shRNA control (3 replicates, merged) MONOMAC-6 samples plotted against minimum read coverage threshold. Correlation strength begins to approach 1.0 at higher thresholds, indicating that stringent thresholding is required for accurate m^{6}A stoichiometry calculation. At a threshold of 50, R^2 = 0.973. Note that for individual scatter plots in the main figures, the Pearson correlation coefficient (r) is reported, whereas here we report R^2 to show the proportion of variance explained.

(B) Upper and lower limits of agreement (LoA) between control samples plotted against minimum read coverage threshold. Limits of agreement were calculated by Bland-Altman analysis and demonstrate reduced variance in m^{6}A stoichiometry measurements with higher thresholding. The mean per-site difference at each threshold is plotted as a grey, dashed line, demonstrating that increased thresholding reduces variance while the mean remains unaffected. At a threshold of 50, 95% of m^{6}A differences between controls are expected to fall between –0.066 and 0.059.

(C) Cumulative distribution plot of per-site m^{6}A stoichiometry differences (Δm^{6}A) between the above control samples for a series of minimum read coverage thresholds. All curves are centered near zero, indicating high concordance between controls. Increasing the read threshold produces steeper slopes and narrower distributions, reflecting reduced variance at higher read depths. The curve corresponding to a threshold of 50 is highlighted in red.

(D) Number of shared, analyzable sites between control samples plotted against read coverage threshold. The number of sites decreased sharply as the threshold increased from zero, reflecting the exclusion of many low-coverage sites, and then began to plateau at higher thresholds as the remaining sites were supported by sufficient coverage. Paired with the previous analyses, we chose an optimal threshold of 50 for subsequent analyses to retain as many analyzable sites as possible. At a threshold of 50, the control samples had 38,018 shared sites.

(E) Scatter plot comparing site-specific m^{6}A stoichiometry from the "All Context" basecaller between NT-shRNA control and DMSO control MONOMAC-6 cells (1 replicate each, n = 116304 sites, ≥50 reads). Pearson's r = 0.98, p < 2e−16. Linear regression line, $y = 1.02x + 0.000119$. We basecalled the raw data with the "All Context" basecaller model to assess non-DRACH m^{6}A sites. The resulting scatter comparison closely matches the results from the DRACH context basecaller.

(F) Bland–Altman analysis comparing m^{6}A stoichiometry measurements between control conditions. Each point represents the difference between paired measurements (DMSO control – NT-shRNA control) plotted against their mean (n = 38018 sites). The solid horizontal line indicates the mean difference (bias = –0.0034; 95% CI: –0.0037 to –

bioRxiv preprint doi: https://doi.org/10.1101/2025.10.22.681652; this version posted October 23, 2025. The copyright holder for this preprint (which was not certified by peer review) is the author/funder, who has granted bioRxiv a license to display the preprint in perpetuity. It is made available under aCC-BY 4.0 International license.

0.0031), while the dashed lines represent the limits of agreement (–0.066 to 0.059; 95% CIs: –0.0666 to –0.0655 and 0.0587 to 0.0598, respectively). This plot serves as a basis for the normal variance to be expected between two controls at a threshold of 50 reads minimum.

bioRxiv preprint doi: https://doi.org/10.1101/2025.10.22.681652; this version posted October 23, 2025. The copyright holder for this preprint (which was not certified by peer review) is the author/funder, who has granted bioRxiv a license to display the preprint in perpetuity. It is made available under aCC-BY 4.0 International license.

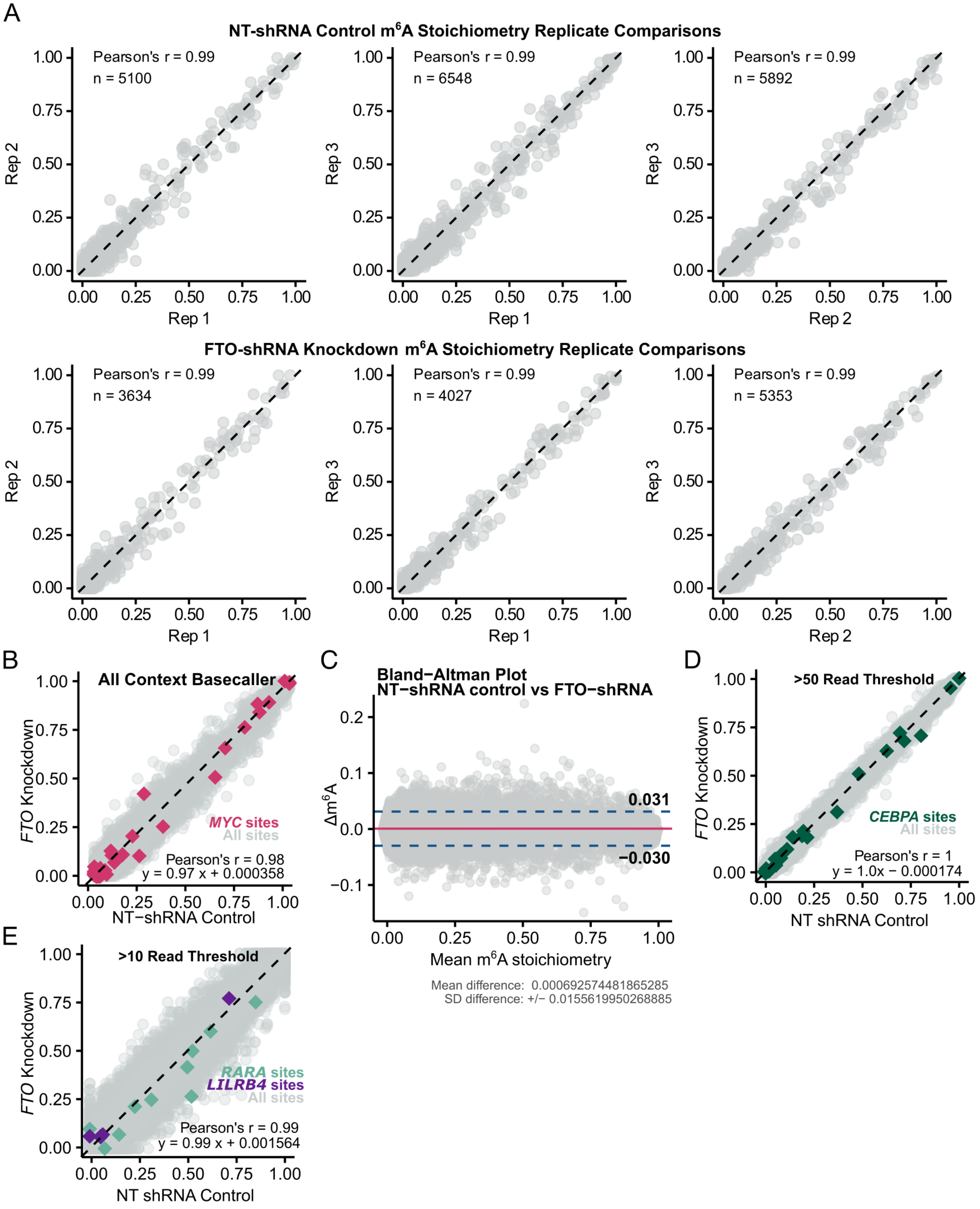

bioRxiv preprint doi: https://doi.org/10.1101/2025.10.22.681652; this version posted October 23, 2025. The copyright holder for this preprint (which was not certified by peer review) is the author/funder, who has granted bioRxiv a license to display the preprint in perpetuity. It is made available under aCC-BY 4.0 International license.

Figure S2. Related to Figure 3.

(A) Pairwise scatter plots comparing per-site m^{6}A stoichiometry calculations between three biological replicates (≥50 reads). NT-shRNA control replicates are compared on the top panel, and FTO-shRNA knockdown replicates are compared on the bottom panel. Pearson's correlation coefficients (r) and number of shared sites (n) are reported in each comparison.

(B) Scatter plot comparing site-specific m^{6}A stoichiometry from the "All Context" basecaller between FTO knockdown and NT-shRNA control MONOMAC-6 cells (1 replicate each, n = 109968 sites, ≥50 reads). *MYC* sites are highlighted in pink (n = 84 sites). Pearson's r = 0.98, $p < 2e{-16}$. Linear regression line, $y = 0.97x + 0.000358$. To ensure that we are not missing any potentially FTO-regulated sites that lie outside of the DRACH consensus site context, we also basecalled our raw data using the "All Context" Dorado basecaller (inosine_m6A), in which every A nucleotide is assessed for modification probabilities. This result is very similar to the DRACH motif basecaller (see **Figure 3A**), with no sites exhibiting increased m^{6}A stoichiometry outside the bounds of normal variability for the "All Context" basecaller (see **Figure S1E**).

(C) Bland–Altman analysis comparing m^{6}A stoichiometry measurements between FTO knockdown and control. Each point represents the difference between paired measurements (NT-shRNA control – FTO-shRNA) plotted against their mean (n = 30880 sites). The solid horizontal line indicates the mean difference (bias = 0.00069; 95% CI: 0.00052 to 0.00087), while the dashed lines represent the limits of agreement (–0.030 to 0.031; 95% CIs: –0.0301 to –0.0295 and 0.0309 to 0.0315, respectively).

(D) Scatter plot comparing site-specific m^{6}A stoichiometry between FTO knockdown and NT-shRNA control MONOMAC-6 cells (n = 29729 sites, ≥50 reads). *CEBPA* sites highlighted in green (n = 26 sites). Pearson's r = 1, $p < 2e{-16}$. Linear regression line, $y = 1.0x + -0.000174$.

(E) Scatter plot with relaxed thresholding comparing site-specific m^{6}A stoichiometry between FTO knockdown and NT-shRNA control MONOMAC-6 cells (n = 89200 sites, ≥10 reads). *RARA* sites are highlighted in teal (n = 10 sites), and *LILRB4* sites are highlighted in purple (n = 3 sites). Pearson's r = 0.99, $p < 2e{-16}$. Linear regression line, $y = 0.99x + 0.001564$.

bioRxiv preprint doi: https://doi.org/10.1101/2025.10.22.681652; this version posted October 23, 2025. The copyright holder for this preprint (which was not certified by peer review) is the author/funder, who has granted bioRxiv a license to display the preprint in perpetuity. It is made available under aCC-BY 4.0 International license.

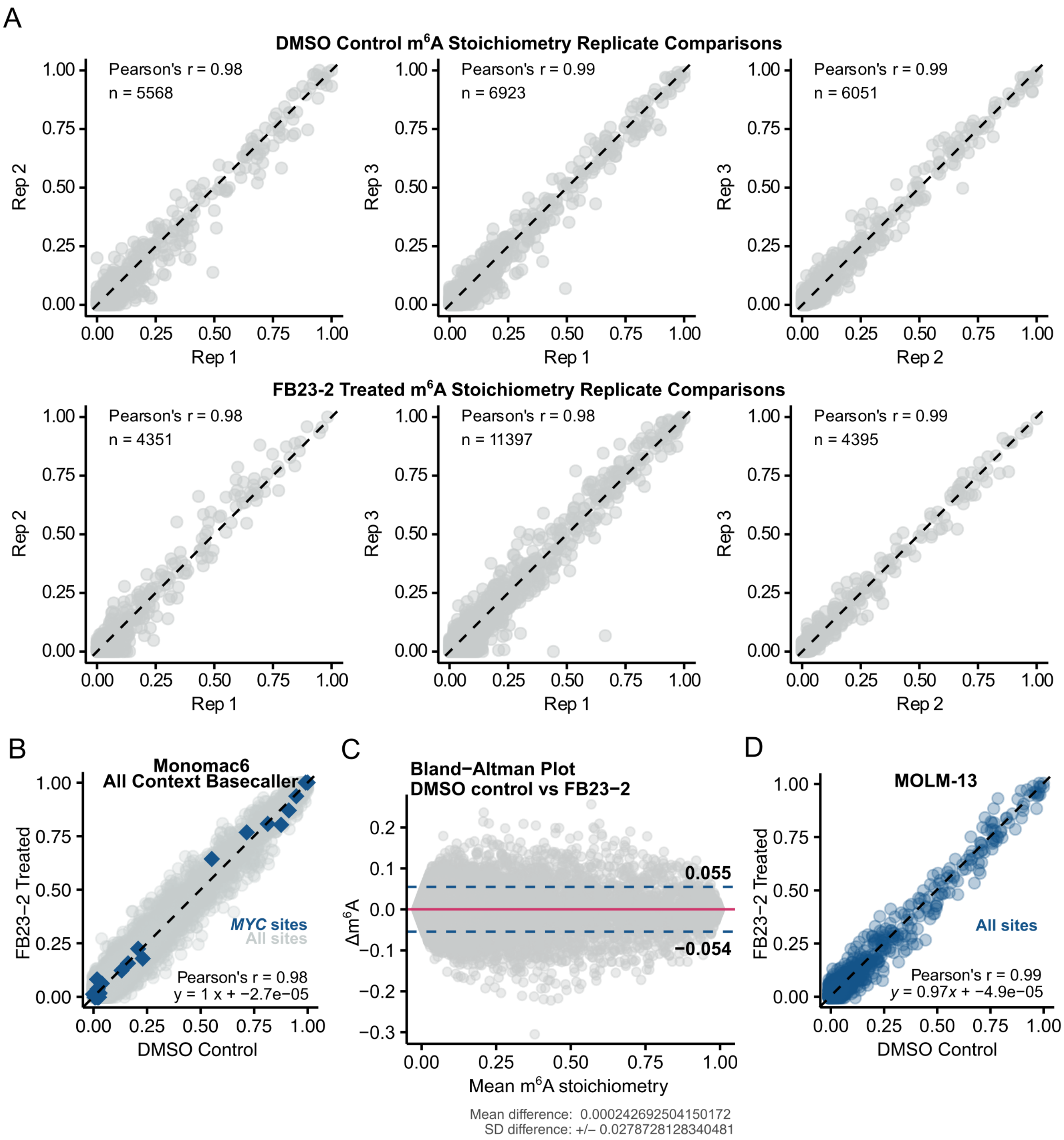

bioRxiv preprint doi: https://doi.org/10.1101/2025.10.22.681652; this version posted October 23, 2025. The copyright holder for this preprint (which was not certified by peer review) is the author/funder, who has granted bioRxiv a license to display the preprint in perpetuity. It is made available under aCC-BY 4.0 International license.

Figure S3. Related to Figure 4.
(A) Pairwise scatter plots comparing per-site m^{6}A stoichiometry calculations between three biological replicates (≥50 reads). DMSO-treated control replicates are compared on the top panel, and FB23-2-treated replicates are compared on the bottom panel. Pearson's correlation coefficients (r) and number of shared sites (n) are reported in each comparison.
(B) Scatter plot comparing site-specific m^{6}A stoichiometry from the "All Context" basecaller between FB23-2-treated and DMSO-treated control MONOMAC-6 cells (1 replicate each, n = 102828 sites, ≥50 reads). *MYC* sites are highlighted in blue (n = 35 sites). Pearson's r = 0.98, p < 2e−16. Linear regression line, $y = 1.0x – 0.000027$. Similar to the knockdown experiment, we basecalled the raw data with the "All Context" basecaller model to assess non-DRACH m^{6}A sites. The resulting scatter comparison of FB23-2-treated and DMSO-treated control m^{6}A sites shows no sites exhibiting increased m^{6}A stoichiometry. Again, the scatter plot closely matches the control comparisons for the inosine_m6A basecaller (see **Figure S1E**).
(C) Bland–Altman analysis comparing m^{6}A stoichiometry measurements between FB23-2-treated and control samples. Each point represents the difference between paired measurements (DMSO control – FB23-2-treated) plotted against their mean (n = 31324 sites). The solid horizontal line indicates the mean difference (bias = 0.00024; 95% CI: -0.000066 to 0.00055), while the dashed lines represent the limits of agreement (–0.054 to 0.055; 95% CIs: –0.0549 to –0.0539 and 0.0543 to 0.0554, respectively).
(D) Scatter plot comparing site-specific m^{6}A stoichiometry between FB23-2-treated and DMSO-treated MOLM-13 cells (1 replicate each, n = 3199 sites, ≥50 reads). Pearson's r = 0.99, p < 2e-16. Linear regression line, $y = 0.97x – 0.000049$.

bioRxiv preprint doi: https://doi.org/10.1101/2025.10.22.681652; this version posted October 23, 2025. The copyright holder for this preprint (which was not certified by peer review) is the author/funder, who has granted bioRxiv a license to display the preprint in perpetuity. It is made available under aCC-BY 4.0 International license.

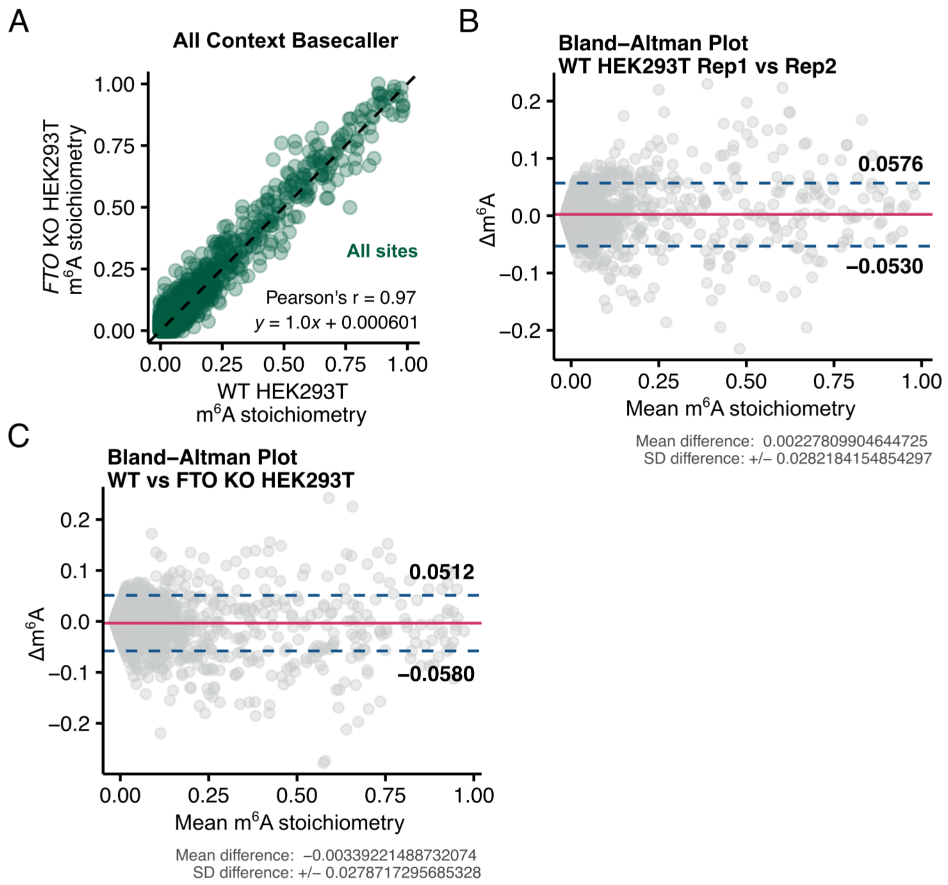

bioRxiv preprint doi: https://doi.org/10.1101/2025.10.22.681652; this version posted October 23, 2025. The copyright holder for this preprint (which was not certified by peer review) is the author/funder, who has granted bioRxiv a license to display the preprint in perpetuity. It is made available under aCC-BY 4.0 International license.

Figure S4. Related to Figure 5.

(A) Scatter plot comparing site-specific m^6A stoichiometry between *FTO* WT and *FTO* KO HEK293T cells basecalled with the inosine_m6A "All Context" Dorado model (n = 21716 sites). Pearson's r = 0.97. Linear regression line, $y = 1.0x + 0.000601$. Again, we assessed potential FTO-regulated sites outside of the DRACH motif context by basecalling the HEK293T raw data with the "All Context" Dorado model. The resulting scatter plot produced very similar results to the DRACH motif basecaller, with no sites exhibiting increased m^6A stoichiometry outside the bounds of normal variability.

(B) Bland–Altman analysis comparing m^6A stoichiometry measurements between replicates of WT HEK293T samples. Each point represents the difference between paired measurements plotted against their mean (n = 3251 sites). The solid horizontal line indicates the mean difference (bias = 0.00228; 95% CI: 0.00131 to 0.00325), while the dashed lines represent the limits of agreement (–0.0530 to 0.0576; 95% CIs: –0.0547 to –0.0514 and 0.0559 to 0.0592, respectively).

(C) Bland–Altman analysis comparing m^6A stoichiometry measurements between *FTO* KO and WT HEK293T samples. Each point represents the difference between paired measurements (WT – *FTO* KO) plotted against their mean (n = 4393 sites). The solid horizontal line indicates the mean difference (bias = –0.00339; 95% CI: –0.00422 to –0.00257), while the dashed lines represent the limits of agreement (–0.0580 to 0.0512; 95% CIs: –0.0594 to –0.0566 and 0.0498 to 0.0526, respectively).

bioRxiv preprint doi: https://doi.org/10.1101/2025.10.22.681652; this version posted October 23, 2025. The copyright holder for this preprint (which was not certified by peer review) is the author/funder, who has granted bioRxiv a license to display the preprint in perpetuity. It is made available under aCC-BY 4.0 International license.

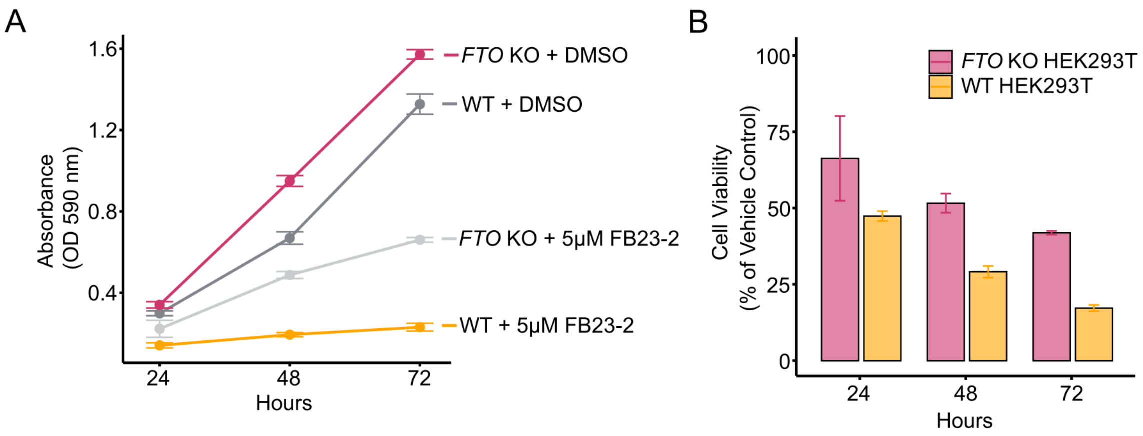

bioRxiv preprint doi: https://doi.org/10.1101/2025.10.22.681652; this version posted October 23, 2025. The copyright holder for this preprint (which was not certified by peer review) is the author/funder, who has granted bioRxiv a license to display the preprint in perpetuity. It is made available under aCC-BY 4.0 International license.

Figure S5. Related to Figure 6.

(A and B) MTT assay cell proliferation and cell viability time courses in *FTO* WT and KO HEK293T cells treated with DMSO or 5 μM FB23-2 (6 replicates each). Cell proliferation was negatively affected by FB23-2 in both *FTO* WT and KO samples **(A)**. Cell viability showed a reduction in both wild-type and FTO-depleted HEK293T cells after drug treatment, with cells lacking FTO showing slightly higher viability **(B)**. Note that DMSO-treated samples reached 100% confluency at 72 hours. Error bars represent mean ± standard error of the mean.
